# Uncovering the roles of DNA hemi-methylation in transcriptional regulation using MspJI-assisted hemi-methylation sequencing

**DOI:** 10.1093/nar/gkae023

**Published:** 2024-01-23

**Authors:** Xiong Xiong, Hengye Chen, Qifan Zhang, Yangying Liu, Chenhuan Xu

**Affiliations:** CAS Key Laboratory of Genome Sciences and Information, Beijing Institute of Genomics, Chinese Academy of Sciences, Beijing 100101, China; China National Center for Bioinformation, Beijing 100101, China; CAS Key Laboratory of Genome Sciences and Information, Beijing Institute of Genomics, Chinese Academy of Sciences, Beijing 100101, China; China National Center for Bioinformation, Beijing 100101, China; CAS Key Laboratory of Genome Sciences and Information, Beijing Institute of Genomics, Chinese Academy of Sciences, Beijing 100101, China; China National Center for Bioinformation, Beijing 100101, China; University of Chinese Academy of Sciences, Beijing 100049, China; CAS Key Laboratory of Genome Sciences and Information, Beijing Institute of Genomics, Chinese Academy of Sciences, Beijing 100101, China; China National Center for Bioinformation, Beijing 100101, China; University of Chinese Academy of Sciences, Beijing 100049, China; CAS Key Laboratory of Genome Sciences and Information, Beijing Institute of Genomics, Chinese Academy of Sciences, Beijing 100101, China; China National Center for Bioinformation, Beijing 100101, China; University of Chinese Academy of Sciences, Beijing 100049, China

## Abstract

Hemi-methylated cytosine dyads widely occur on mammalian genomic DNA, and can be stably inherited across cell divisions, serving as potential epigenetic marks. Previous identification of hemi-methylation relied on harsh bisulfite treatment, leading to extensive DNA degradation and loss of methylation information. Here we introduce Mhemi-seq, a bisulfite-free strategy, to efficiently resolve methylation status of cytosine dyads into unmethylation, strand-specific hemi-methylation, or full-methylation. Mhemi-seq reproduces methylomes from bisulfite-based sequencing (BS-seq & hpBS-seq), including the asymmetric hemi-methylation enrichment flanking CTCF motifs. By avoiding base conversion, Mhemi-seq resolves allele-specific methylation and associated imprinted gene expression more efficiently than BS-seq. Furthermore, we reveal an inhibitory role of hemi-methylation in gene expression and transcription factor (TF)–DNA binding, and some displays a similar extent of inhibition as full-methylation. Finally, we uncover new hemi-methylation patterns within *Alu* retrotransposon elements. Collectively, Mhemi-seq can accelerate the identification of DNA hemi-methylation and facilitate its integration into the chromatin environment for future studies.

## Graphical Abstract

**Graphical Abstract:**
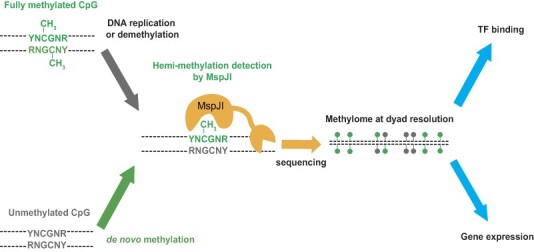

## Introduction

DNA methylation is an important epigenetic modification in which a methyl group is covalently ligated to a nucleotide by DNA methyltransferases (DNMTs). In mammals, DNA methylation mainly occurs at cytosines within CpG dinucleotides and plays crucial roles in gene expression regulation. At the same CpG dyad, the cytosines on the two DNA strands usually have the same methylation status (both methylated or unmethylated). However, during DNA replication, demethylation, or *de novo* methylation, hemi-methylated CpG dyads are generated in the genome (1,2). Recent work suggests that hemi-methylation is abundant and dynamic, and some of them could be stably inherited across cell divisions, serving as potential epigenetic marks during embryonic development (1,3,4). However, an efficient method for identifying hemi-methylation and a systematic study on its function are still lacking.

In the past decades, to understand the roles of DNA methylation, various methylation mapping methods have been developed. These methods can be classified into four groups based on their principles: (i) base conversion, (ii) affinity pull-down, (iii) single-molecule sequencing and (iv) methyl-sensitive restriction enzyme digestion. The most commonly used base conversion method is called bisulfite conversion in which unmethylated cytosines are converted to uridines. This method coupled with next-generation sequencing (bisulfite sequencing, BS-seq) can generate cytosine methylomes at single-base resolution. In addition, a modified BS-seq method named hairpin BS-seq (hpBS-seq) is able to resolve hemi-methylated cytosine dyads. However, after bisulfite conversion, DNA is fragmented and become AT-rich, resulting in DNA breakdown, increase of PCR bias, and loss of genomic variant information. Affinity pull-down methods utilize antibodies or methyl-CpG-binding domain (MBD) to enrich methylated DNA (5,6). These methods specifically capture highly methylated regions but cannot provide single CpG resolution. Recently, the development of third-generation sequencing techniques, Pacbio and Nanopore, allows direct reading of DNA modifications on single DNA molecules at single base and single base-pair resolution, respectively (7–10). But the cost of these methods is much higher than methods based on next generation sequencing (NGS), and special algorithms are required to read 5-methylcytosine signals. Methyl-sensitive restriction enzymes capture DNA methylation by specifically cleaving DNA near methylated recognition sites. A few methyl-dependent restriction enzymes, such as MspJI, have been used to develop DNA methylation mapping methods (11–14). These methods are normally sensitive, cost-efficient, and easy to perform. However, they are unable to resolve hemi-methylation at single dyad resolution.

Here, in this work, to systematically investigate the role of DNA hemi-methylation in transcriptional regulation, we renovated previous MspJI-based methods and developed a bisulfite-free method named MspJI-assisted hemi-methylation sequencing (Mhemi-seq). In this method, genomic DNA is digested by MspJI which cuts at 9/13 bp downstream of methylated CNNR sites. Sequencing adapters are directly ligated to the digested fragments followed by NGS, and the position of mCNNR was deduced by the cutting edges of sequencing reads. Our results reveal that Mhemi-seq accurately resolves hemi-methylation and efficiently identifies allele-specific DNA methylation. Furthermore, we discover that hemi-methylation can inhibit transcription and TF binding in a similar extent as full-methylation, suggesting hemi-methylation may be sufficient to exert the inhibitory roles DNA methylation plays in transcriptional regulation.

## Materials and methods

### Reagents

The following items were key reagents and kits used in this study: GSK-3484862 (HY-135146, MedChemExpress), NEB T4 ligase (M0202L, NEB), MspJI (R0661S, NEB), EZ DNA Methylation-Lightning Kit (D5030, Zymo), anti-Rabbit IgG antibody (3418S, Cell Signaling Technology), anti-CTCF antibody (2729S, Cell Signaling Technology) and HiFi HotStart Uracil + DNA Polymerase (KK2801, Roche).

### Cell culture

GM12878 cells were cultured in RPMI (Vivacell) supplied with 10% Fetal bovine serum (Biological Industries) under 5% CO_2_ at 37°C. For GSK-3484862 (HY-135146, MedChemExpress) treatment, GM12878 cells were plated in 10-cm dishes at a cell density of ∼3 × 10^6^/dish. The next day, cells were treated with 0.1% DMSO or 5 μM GSK-3484862 for four consecutive days. On Day 3 of treatment, the cells were subcultured and DMSO (D2650, Sigma) or GSK-3484862 were replenished.

### *In vitro* MspJI digestion assay

45 μl of 100 μM FragA1 oligo (Supplementary Table S1), 45 μl of 100 μM FragA2 oligo (Supplementary Table S1), and 10 μl of 10× Annealing Buffer (1 M potassium acetate; 300 mM HEPES–KOH, pH7.5) were mixed followed by 95°C denaturation for 3 min. The reaction was slowly cooled down to anneal FragA1 and FragA2, and generate double-stranded Frag1. Double-stranded Frag2, Frag_me, and Frag_hemi were generated by annealing FragB1 and FragB2, Frag_mC1 and Frag_mC2, and Frag_C1 and Frag_mC2 (Supplementary Table S1), respectively. A ligation reaction was prepared with 1 μl of Frag1, 1 μl of Frag2, 1 μl of Frag_mC1 or Frag_mC2, 2 μl of 10× NEB T4 DNA ligase buffer, 1 μl of NEB T4 ligase (M0202L, NEB) and 14 μl of ddH_2_O. This reaction was incubated at 20–25°C for 2 h, and the 146 bp ligation product was then purified by Ampure beads (A63880, Beckman). The ligation product was digested in a reaction containing 100 ng of this ligation product, 2 μl of 10x rCutSmart buffer (B6004S, NEB), 0.7 μl of 30× Enzyme Activator, 0.3 μl of MspJI (R0661S, NEB), and 15 μl of ddH_2_O at 37°C for 2 h. The digestion products were then assessed using a 20% polyacrylamide gel.

### Genomic DNA extraction

Cells were collected by centrifugation at 4°C for 3 min at 300 ×*g*, washed with 1× PBS (P1022, Solarbio), and resuspend with 600 μl Lysis Buffer (10 mM Tris–HCl, pH 8.0; 10 mM EDTA, pH 8.0; 10 mM NaCl; 0.5% SDS; 50 ng/μl RNase A (R6513, Sigma); 1 μg/μl Proteinase K (3115852001, Roche)). The suspension was incubated at 55°C for 40–60 min with interval mixing. gDNA was extracted by phenol:chloroform:isoamyl alcohol (P3803, Sigma) extraction and ethanol precipitation, and stored at −80°C.

### Mhemi-seq experimental procedures

A 20 μl reaction was prepared using 10–100 ng of genomic DNA, 10x NEB rCutSmart buffer, 30× MspJI activator, and MspJI (2.5 units per 100 ng of DNA). The reaction was incubated at 37°C for 1 h followed by the addition of 1 μl of 100 mM ATP (R0441, Thermo Fisher), 1.5 μl of 45 μM annealed Mhemi-seq adapter (Supplementary Table S1), and 400 Unit of NEB T4 Ligase. The reaction was then incubated at 20–25°C for 2 h. After T4 ligation, 94 μl of AMPure beads (18% PEG, 1:4) was added to the reaction, and the sample was mixed by pipetting and rotated at 20–25°C for 10 min. Supernatant was removed using a magnetic rack, and beads were washed twice with 75% ethanol. Beads were air-dried for 1–3 min to remove residual liquid. DNA was eluted from beads by 23.5 μl H_2_O. The elute was mixed with 25 μl of 2× KAPA HiFi HotStart ReadyMix (KK2602, Roche) and 1.5 μl of 10 μM sequencing primer mix (Supplementary Table S1). PCR reaction was run using the following cycling condition: 95°C 3 min for 1 cycle; 98°C 20 s, 60°C 15 s and 72°C 30 s for 4–8 cycles; and 72°C 1 min for 1 cycle. A test electrophoresis was performed to check the amplified fragment size. A ∼200–500 bp smear should be observed on an agarose gel. PCR product was then cleaned-up by AMPure beads (18% PEG, 1:1.4). The final amount of DNA should be ∼50 ng. The libraries were sequenced using Illumina NovaSeq pair-end sequencing platform. A detailed protocol was provided as supplementary materials (Supplementary File S1).

### hpBS-seq experimental procedures

About 3–10 μg genomic DNA was sonicated with 30–40 cycles of ‘30% Efficiency, 30 s ON, 30 s OFF’ on Bioruptor to reach a 100–200 bp smear. 1 μg of sheared DNA was mixed with 1.5 μl of NEB End Repair Enzyme Mix (E6050L, NEB) and 3 μl of 10× NEB End Repair Reaction Buffer in a 30 μl volumn, and was incubated at room temperature for 30 min. DNA was cleaned-up by AMPure beads (18% PEG, 1:1.8). Next, the A-tailing reaction is done by using 1 μl of *Taq* DNA Polymerase (EP0402, Thermo Fisher) and 3 μl of 10× A-tailing mix (100 μl of 10× ThermoPol Reaction Buffer (B9004S, NEB), 1 μl of 100 mM dATP) in a 30 μl volumn at 37°C for 30 min. DNA was cleaned-up by AMPure beads (18% PEG, 1:1.8). The ligation step was done by using 0.8 μl of T4 DNA Ligase (EL0012, Thermo Fisher), 3 μl of 10× T4 DNA Ligase Buffer (Thermo Fisher), 0.2 μl of 9 μM hairpin adapter (Supplementary Table S1) and 0.8 μl of Illumina TruSeq DNA PCR-Free adapter (20015960, Illumina) in a 30 μl reaction at room temperature for 2 h. DNA was cleaned-up by AMPure beads (18% PEG, 1:1.4). The DNA was incubated with Dynabeads Streptavidin C1 beads (65002, Thermo Fisher) on a rotor for 30 min at room temperature, and was washed separately by 500 μl of 1× BW Buffer according to the manufacturer's instructions, 200 μl of 150 mM NaOH with 0.01% Tween-20 and 200 μl of 10 mM Tris–HCl, pH 8.0. The beads were resuspended in 95% formamide and 10 mM EDTA pH 8.2, and incubated at 95°C for 3 min. Supernatant was collected and was ethanol precipitated and bisulfite converted with the EZ DNA Methylation-Lightning Kit (D5030, Zymo). PCR amplification was done with HiFi HotStart Uracil + DNA Polymerase (KK2801, Roche) and was run using the following cycling condition: 95°C 3 min for 1 cycle; 98°C 20 s, 60°C 15 s and 72°C 30 s for 11–13 cycles; and 72°C 1 min for 1 cycle. PCR product was then cleaned-up by AMPure beads (18% PEG, 1:1) and the final amount of DNA should be ∼50 ng. The libraries were sequenced using Illumina NovaSeq pair-end sequencing platform.

### ChIP-seq experimental procedures

Cells were crosslinked by 1% formaldehyde (F8775, Sigma) for 10 min at room temperature. The reaction was terminated by adding 2.5 M glycine (V900144, Sigma) to a final concentration of 125 mM. After being washed with PBS, the cells were resuspended in 1 mL of Lysis Buffer (5 mM PIPES, pH 8.0; 85 mM KCl; 0.5% NP-40; 10% glycerol) and rotated at 4°C for 15 min. After centrifugation, the chromatin fraction was resuspended in 200 μl of RIPA Buffer (1× PBS; 1% NP-40; 0.5% sodium deoxycholate; 0.1% SDS) and sonicated into 200–400 bp fragments using Bioruptor. Before Immunoprecipitation, the anti-Rabbit IgG antibody (3418S, Cell Signaling Technology) and anti-CTCF antibody (2729S, Cell Signaling Technology) were incubated with pre-washed Dynabeads Protein A (10002D, Thermo Fisher) at 4°C for 4–6 h or overnight. Of the sheared chromatin, 5% was set aside as Input, and the rest was immunoprecipitated with the Protein A-IgG for 30 min at 4°C and the Protein A-CTCF for 4–6 h at 4°C. The beads were sequentially washed five times with 1 ml of LiCl Wash Buffer (100 mM Tris–HCl pH 7.5; 500 mM LiCl; 1% NP-40; 1% sodium deoxycholate). Chromatin complexes were eluted by incubation with 200 μl of Elution Buffer (50 mM Tris–HCl, pH 8.0; 10 mM EDTA, pH 8.0; 1% SDS) at 65°C for 20 min with periodic gentle vortex and supernatant was removed using a magnetic rack. 190 μl of Elution Buffer was added to the Input, and the supernatant and Input were de-crosslinked at 65°C for 6 h. Then, 200 μl of TECa buffer (10 mM Tris–HCl, pH 8.0; 1 mM EDTA, pH 8.0; 10 mM CaCl_2_) and 10 μl of proteinase K were added to the reactions, which were incubated at 55°C for 30 min. DNA was extracted with phenol:chloroform and precipitated with ethanol. The ChIP-seq libraries were constructed using the following steps: (a) End Repair is accomplished by using the NEBNext End Repair Module at RT for 30 min. (b) A-tailing is accomplished by using 1 μl of *Taq* DNA Polymerase and 3 μl of 10× A-tailing mix (100 μl of 10× ThermoPol Reaction Buffer, 1 μl of 100 mM dATP) at 37°C for 30 min. (c) Adapter ligation is accomplished by using 0.8 μl of T4 DNA Ligase of Thermo Fisher, 0.6 μl of 45 μM Illumina adapters at room temperature for 2 h. PCR reaction was run using the following cycling condition: 95°C 3 min for 1 cycle; 98°C 20 s, 60°C 15 s, and 72°C 30 s for 4–8 cycles; and 72°C 1 min for 1 cycle. PCR product was then cleaned-up by AMPure beads (18% PEG, 1:1). The libraries were sequenced using Illumina NovaSeq pair-end sequencing platform.

### Mhemi-seq data preprocessing

**Figure 1.**
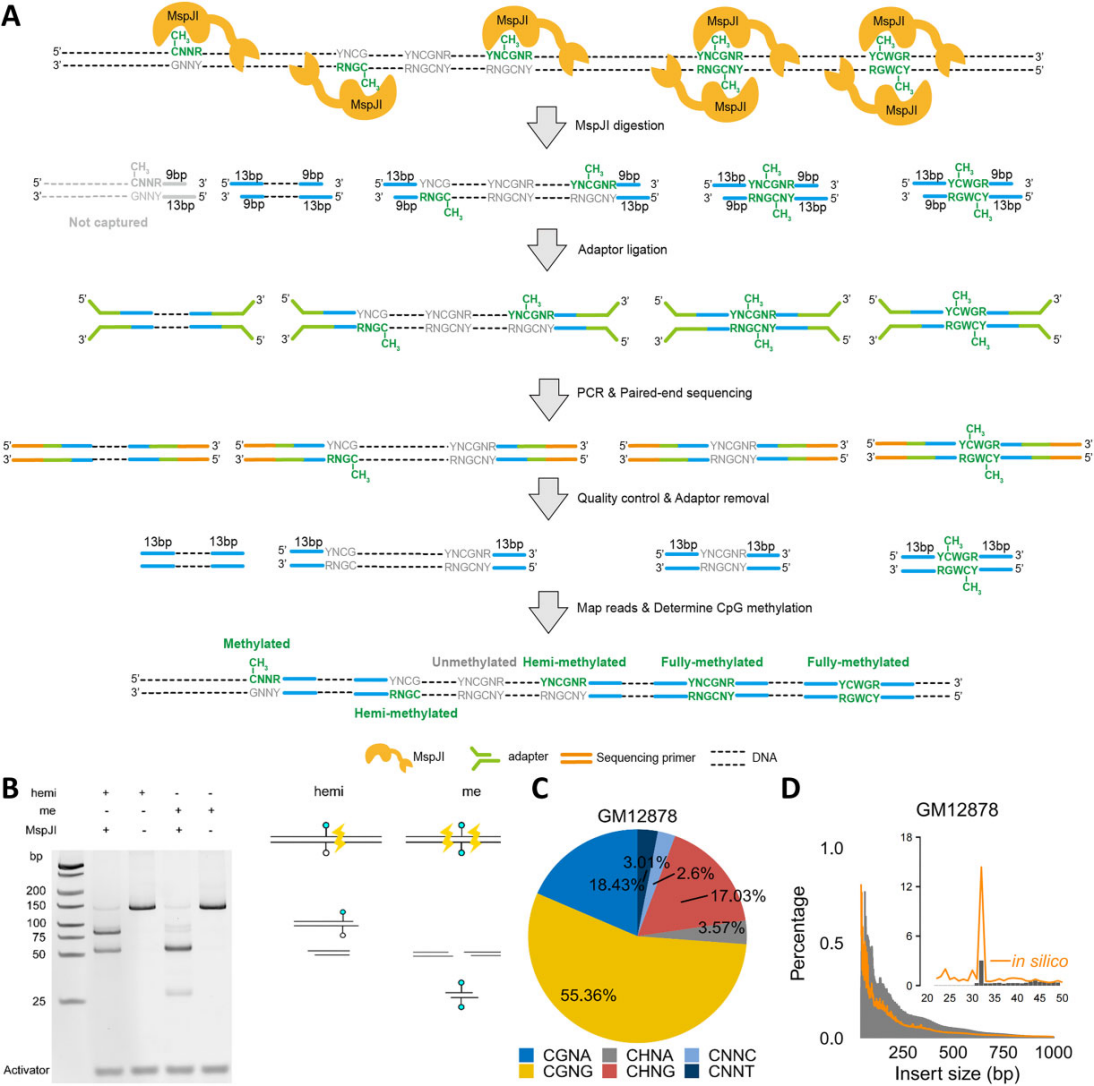
Proof of principles and technical statistics of Mhemi-seq. **(A)** Scheme of strand-specific mCNNR, mCpG, and mCWG detection using Mhemi-seq. **(B)** MspJI cut DNA oligonucleotides in different manners according to their methylation status. Reaction mix was run on a 20% polyacrylamide TBE gel and visualized by GelRed staining. **(C)** Motif analysis reveals the specificity of MspJI on CNNR sites in GM12878. **(D)** Length distribution of inserts in Mhemi-seq library (grey histogram) versus the length distribution simulated using BS-seq results (orange line).

The 150 bp paired-end reads were trimmed by fastp (15) using following parameters: fastp –umi –umi_loc per_read –umi_len2 –correction -q 20 -e 20 -l 30. Trim Galore wrapped around Cutadapt and FastQC was applied for further adapter and quality trimming with default parameters (16). Trimmed reads were then aligned to the human genome reference hg38 by bowtie2 with -X 1000 –no-unal –no-mixed –no-dicordant. Samtools was used to remove low mapping quality (MAPQ < 10) for alignments with size over 100 bp. Duplicated reads were removed by considering genomic position and UMI due to the low complexity of 32 bp fragments. Alignment information in the bam file was converted to fragment position information by bamToBed in bedtools package. Based on the position of each fragment and the CGNR motif in the upstream and downstream, the methylation status of the CGNR and YNCGNR motifs were determined (Figure 1A). The methylation status at CHNR and YCWGR motifs was determined by the same principle. Only motifs covered by more than four reads were used in the statistical analysis. Codes for Mhemi-seq data processing are available at https://github.com/xiongxionghhh/Mhemi-seq.

### Normalization of Mhemi-seq data

The four methylation status, unme, hemiW, hemiC and me, were normalized based on the following equations: (i) M_exp_= M_real_∗E_Watson_*E_Crick_*E_pu_, (ii) W_exp_= W_real_+ M_real_*E_Watson_*(1-E_Crick_), (iii) C_exp_= C_real_+ M_real_*(1-E_Watson_)*E_crick_ and (iv) U_exp_= U_real_+ M_real_*(1-E_Watson_)*(1 – E_Crick_). In these equations, *M*, *W*, *C* and *U* represent the count of CpGs that are fully methylated, hemi-methylated at Watson and Crick strand, and unmethylated, respectively. The footnotes *exp* and *real* represent the counts captured by Mhemi-seq and the theoretical count, respectively. *E_Watson_* and *E_Crick_* stand for the cutting efficiency at Watson and Crick strands of the YNCGNR motif. These efficiencies were determined by the cutting efficiency at CGNA and CGNG. *E_pu_* is the purification efficiency of 32 bp fragments. Codes and example input are available at https://github.com/HengyeChen/Mhemi_normalization.

### hpBS-seq data preprocessing

The 150 bp paired-end reads were initially trimmed using cutadapt (3.7) to eliminate any remaining hairpin linker sequence and adapter sequence. Subsequently, the mate 1 and mate 2 reads were individually aligned to the human genome (hg38) using bismark (0.23.0) with bowtie2 (2.4.5) (17,18). The alignment score was controlled by applying the bowtie2 option –score-min L,0,-0.4, and mapping uniqueness was ensured by selecting alignments with a mapping quality MAPQ > 10 using Samtools (1.16.1) (19). Duplicate reads were removed through bismark, and methylation status of each dyad was extracted from specific pairs of adjacent cytocines on both Watson and Crick strands using bedtools (2.30.0) (20). The bisulfite conversion rate of each library was calculated according to the unmethylated cytosines on the hairpin linker.

### ChIP-seq data preprocessing

The 150 bp paired-end reads were aligned to the human genome (hg38) using bowtie2 with an alignment score controlled by applying –score-min L,0,-0.4 option. Mapping uniqueness was ensured by selecting alignments with a mapping quality MAPQ > 10 using Samtools. The data was normalized with reads per million (RPM) and the BigWig files were generated using bedGraphToBigWig(v4). A matrix underlying line plot was created utilizing deepTools(3.5.0) (21).

### CTCF motifs annotation

A list of 113043 CTCF motifs ever detected as occupied in at least one cell type was compiled from all available CTCF ChIP-seq experiments in human cell lines deposited in ENCODE as previously described (22). For calculating the reads per million (RPM) value of ChIP-seq data, a global division of the read density with the total number of alignments (in million) was done. For annotating the CTCF RPM value of CTCF motifs, the mean RPM value from a 100 bp window surrounding the CTCF motif center base was calculated.

### CTCF CpG dyad methylation status assignment

The G-rich strand of consensus CTCF motif was defined as the motif strand (23). The length of CTCF motif was defined as 19 bp according to the CTCF motif (MA0139.1) from JASPAR (24). The methylation status of dyads (≥3 counts) obtained from hpBS-seq, was assessed based on the most predominant status out of four statuses (unme, hemi-Watson, hemi-Crick, me). Dyads for which methylation status could not be definitively determined were excluded from the analysis. By aligning the dyad positions of CTCF with those derived from hpBS-seq, a classification of their methylation status was established.

### *In silico* digestion of genome by MspJI

For CpGs covered by more than four BS-seq reads, the methylation frequency at each CpG was calculated using the corresponding reads. For other CpGs, the methylation frequency of each CpG was determined by the average methylation frequency. The methylation frequency at each CGNR was used to determine the cutting probability at this site. Based on this probability, 0 or 1 was stochastically assigned to each cutting site, representing the site uncut or cut by MspJI, respectively. The final simulation was conducted using these 0 and 1 values, and the distance between two neighboring 1 sites was defined as a fragment length. The lengths of all fragments were then plotted in a histogram to show the distribution.

### Comparison of Mhemi-seq and BS-seq

**Figure 2.**
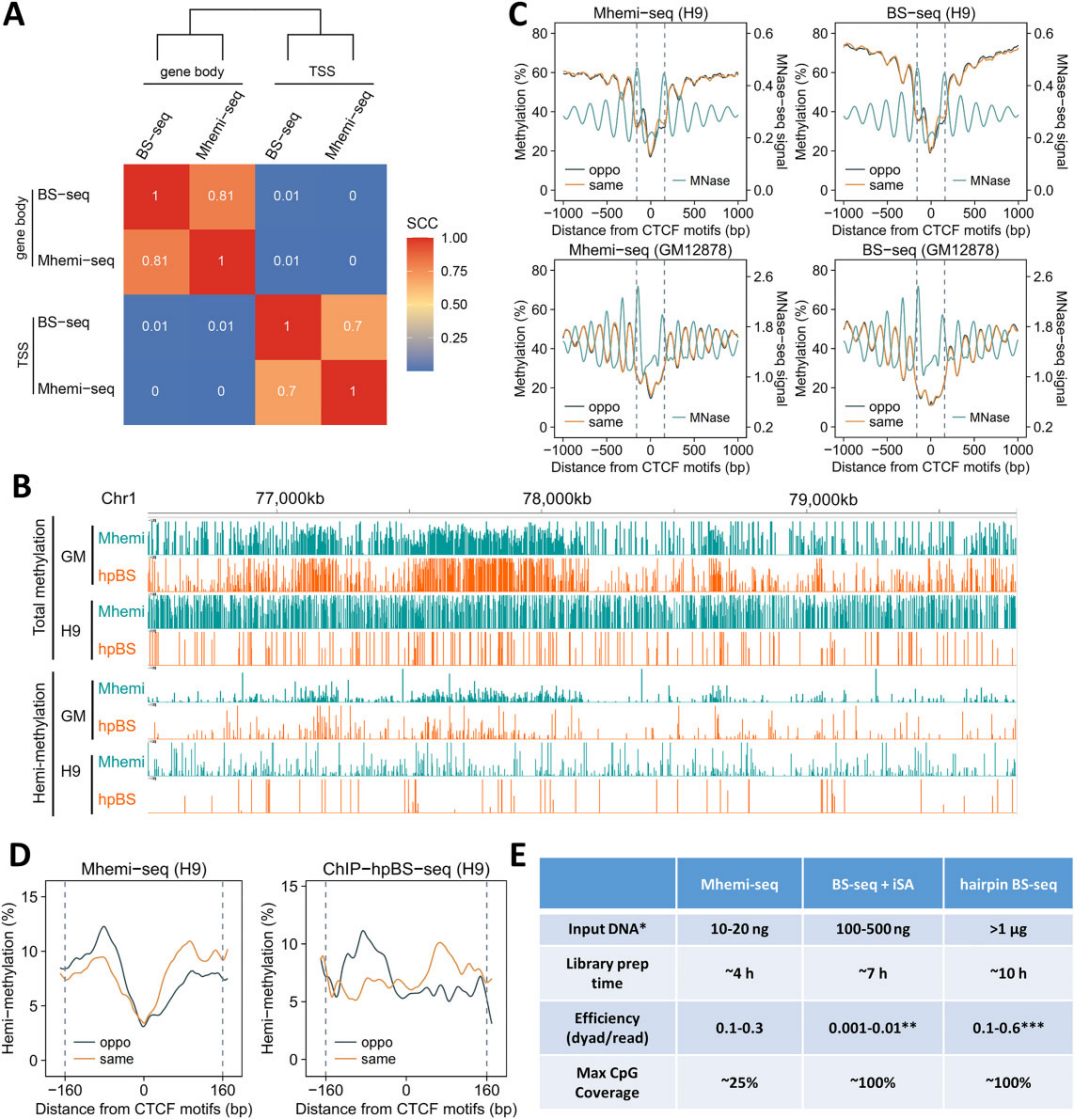
Mhemi-seq reproduces cytosine methylome from BS-seq and dyad methylome from hpBS-seq. **(A)** Spearman's correlation between Mhemi-seq and BS-seq at TSS and gene body in H9 cells. **(B)** Comparison of methylation captured by Mhemi-seq and hpBS-seq in GM12878 and H9 cells. **(C)** Hemi-methylation profiles of Mhemi-seq (left) and BS-seq (right) are consistent near CTCF motifs in H9 and GM12878 cells. Same, oppo, and MNase represent hemi-methylation at motif strand, opposite strand, and MNase-seq signal. **(D)** Mhemi-seq reproduces the hemi-methylation pattern mapped by ChIP-hpBS-seq near CTCF motifs. **(E)** Performance comparison of Mhemi-seq, BS-seq and hpBS-seq in mapping CpG dyads. Efficiency represents how many CpG dyad can be captured per (pair of) sequencing read(s). *Amount for average 5× coverage of mappable cytosines in human genome. **Efficiency highly depends on sequencing depth. ***Efficiency greatly varies across different studies.

To compare Mhemi-seq and BS-seq at CpG level, we first defined the upstream 1.5 kb to downstream 0.5 kb of the TSS and the TSS to TTS of the gene as the TSS region and gene body region, respectively, and then divided each region into 300 bins. bigWigAverageOverBed command was used to calculated methylation level of each bin. Pearson correlation coefficient was used to assess the similarity of two datasets. For correlation related to Figure 2A and Supplementary Figure S2A, we only included upstream 1.5 kb of TSS as TSS regions to avoid overlap, and use Spearman correlation coefficient instead. In addition, single-base resolution methylation map surrounding CTCF binding sites was used to compare Mhemi-seq and BS-seq since relatively stable and apparent hemi-methylation exists on this region. To compare Mhemi-seq and BS-seq at CpG dyad level, hemi-methylation levels with dyad resolution of regions surrounding CTCF binding sites were calculated by Mhemi-seq and BS-seq. Strands were classified by CTCF motif orientation. Furthermore, genomic distribution of CpG dyads was determined using annotatePeaks command in Homer software (25).

### Classification of CTCF motif and PCGF1 bound promoters

CTCF CpG dyad methylation assignment was performed as described above. Dyad methylation levels of the 5th (Dyad 5) and 7th CpG dyad (Dyad 7) were used for CTCF motif classification with these two dyads, respectively. ChIP-seq signal of each motif was calculated using bigWigAverageOverBed command with the parameter: -sampleAroundCenter = 40. PCGF1 bound promoters (1.5 kb upstream to 0.5 kb downstream of the TSS) were classified by the same strategy using CpG dyads in them.

### RNA-seq data processing

Processed RNA-seq datasets in bam format were downloaded from ENCODE (22). Transcript per kilobase million (TPM) of each transcript in a sample was calculated using StringTie (26). Transcript per kilobase million (TPM) of each *Alu* in a sample was calculated by custom script using read count as input obtained from featureCounts (v2.0.1) (27).

### Analysis of allele-specific TF binding, methylation and gene expression

SNPs information of GM12878 was downloaded for allele-specific analysis (28). Reads with paternal and maternal SNPs were separated into paternal and maternal specific alignments, respectively. For BS-seq, as it artificially introduces base conversion (i.e. C to T), C→T SNPs could either be a genuine SNP or rather reflect the methylation state. Thus, all of those confusing SNPs were excluded for alignment separation. For other sequencing, all SNPs were used. Paternal and maternal specific reads were then separately analyzed using the methods described above. Allele-specific peaks were called using AESReadCounter in GATK package (29). Binomial test was performed on SNPs with coverage larger than 10 as previously described (30). A SNP was defined as a bias-SNP if the false discovery rate (FDR) is smaller than 0.05. Genes or peaks with bias-SNPs were defined as allele-specific expressed genes or allele-specific peaks, respectively. Genes with allele-specific expression, TF binding peaks, and methylation were selected and visualized in genome browser (31).

### Statistical analysis

All of the statistical details can be found in the figure legends. Differences were analyzed with several statistical tests, as indicated in figure legends. Error bars represent the mean ± standard deviation.

## Results

### Mhemi-seq efficiently resolves methylation states of single CpG dyads

MspJI recognizes a 5′-mCNNR-3′ motif and cleaves DNA at 9/13 bp downstream of the motif, generating a 4 bp 5′ overhang (14) (Figure 1A). We posited that within a palindromic 5′-YNCGNR-3′ motif, MspJI would independently recognize each of the two cytosines if methylated, and cut at 9/13 bp downstream of the respective motif, giving rise to fragments with a characteristic 32 bp or usually > 50 bp length when the CpG dyad is fully methylated or hemi-methylated, respectively. In turn, the fragment length would instruct us to determine the methylation state of the CpG dyad when the cytosine is within the YNCGNR motif which is located 13 bp away from the sequencing read edge (Figure 1A). In addition, the same principle can be used to determine the hemi-methylation at YCWGR motifs and methylation at CGNR motifs inside or outside reads when only cytosine methylation information is required (Figure 1A).

To prove that MspJI digests DNA as expected, we used MspJI to digest synthesized double-strand DNA molecules containing either a hemi-methylated or fully methylated CpG dyad within the motif YNCGNR. Our results showed that MspJI cut at only one side of the hemi-methylated CpG dyad and generated one 89 bp and one 57 bp fragments, while on the fully methylated template, MspJI cut at both sides of the CpG dyad and generated two 57 bp and one 32 bp fragments (Figure 1B). The result demonstrated that MspJI can independently read the methyl group on each strand and accurately cut at an expected distance downstream of the methylated cytosine. Since previous studies suggest that MspJI prefers to digest CGNG and has star activity on unmethylated DNA (32), to further evaluate the MspJI digestion in Mhemi-seq, we digested synthetic DNA templates with unmethylated, hemi-methylated at Watson or Crick strand, and fully methylated TTCGGG site (Supplementary Figure S1A). Our results showed that unmethylated template was barely digested by MspJI, while ∼97% and ∼80% of mCGGG and mCGAA sites were digested respectively (Supplementary Figure S1B). Thus, in our assay, the star activity of MspJI is not of concern, and the digestion at mCGGG sites is near-complete, but the insufficient digestion at mCGNA sites will lead to under-estimation of the methylation level.

Next, we streamlined the MspJI digestion with NGS library preparation, developed Mhemi-seq, and applied it in B-lymphocyte cell line GM12878, and human embryonic stem cell (hESC) lines H1 and H9. Most DNA fragments (94.6%, 94.8% and 94.8% for GM12878, H1 and H9, respectively) contained the CNNR motifs 13 bp away from the read edges, indicating the high recognition specificity and the cutting distance stringency of MspJI (Figure 1C and Supplementary Figure S1C). Among all sites, the dominant site is CGNR (73.8%, 70.3% and 66.9%), while CHNR (H = A/T/C) accounts for 20.6%, 24.5% and 27.9% in GM12878, H1 and H9 cells, respectively (Figure 1C and Supplementary Figure S1C). This is consistent with the fact that the methylation level at CpG is much higher than at CpH in human somatic cells (33–35). To check the efficiency of Mhemi-seq, we quantified how many CGNR motifs were captured per sequencing read (Supplementary Figure S1D). Our results revealed that about 50–66% of mapped fragments contain CGNR motifs at both sides, and about 0.1–0.3 CpG dyad (YNCGNR) is mapped per read (Supplementary Figure S1D). In addition, we found that 80.1% of the 32 bp fragments contains YNCGNR motifs in the center position, suggesting the high motif fidelity and methylation dependency of MspJI (Supplementary Figure S1E). To further prove that MspJI cut genomic DNA as expected, we simulated the distribution of fragment length generated by MspJI digestion. We found that the fragment length distribution of Mhemi-seq results was similar to the distribution simulated using BS-seq results (Figure 1D and Supplementary Figure S1F). Mhemi-seq exhibits good reproducibility in resolving methylation at CpG and CpG dyad between replicates, especially for regions with very high or low methylation level (Supplementary Figure S1G and S1H). For CpGs with intermediate methylation levels, the batch-to-batch variability is significant, but this variability could be reduced by selecting CpGs with higher coverage or averaging CpG methylation level in 10 kb bins (Supplementary Figure S1H–J). All the above results suggest that Mhemi-seq, as a bisulfite-free method, has the potential to generate base-resolution DNA methylome maps.

### Mhemi-seq reproduces cytosine methylome from BS-seq and dyad methylome from hpBS-seq

To examine if Mhemi-seq can produce quantitative and base-resolved DNA methylomes, we first compared Mhemi-seq results with results from BS-seq, the gold standard of DNA methylation mapping. In general, Mhemi-seq exhibits consistency with BS-seq and hpBS-seq at genome-wide methylation level (Figure 2A, B and Supplementary Figure S2A–E). Particularly, Mhemi-seq reproduced the phasing of cytosine methylation at CCCTC-binding factor (CTCF) binding sites (Figure 2C, D and Supplementary Figure S2F). Our recent study discovered that asymmetric hemi-methylated CpG dyads reside in the two flanking regions of CTCF motifs in pluripotent cells (3). Next, we compared Mhemi-seq data with published CTCF ChIP-hairpinBS-seq results (3). At dyad methylation level, Mhemi-seq displayed a similar hemi-methylation pattern as the ChIP-hairpinBS-seq results at CTCF motifs (Figure 2D and Supplementary Figure S2F), demonstrating that Mhemi-seq is able to accurately resolve methylation state of single CpG dyads. Compared to other mainstream bisulfite-based methylation mapping methods, Mhemi-seq has a simpler procedure, resolves dyad methylation in a similar efficiency with hpBS-seq, but requires much less input DNA, allowing prompt methylation detection in a high-throughput and cost-efficient manner when CpG dyad resolution is required (Figure 2E).

### Mhemi-seq efficiently identifies allele-specific methylation and imprinted gene expression

**Figure 3.**
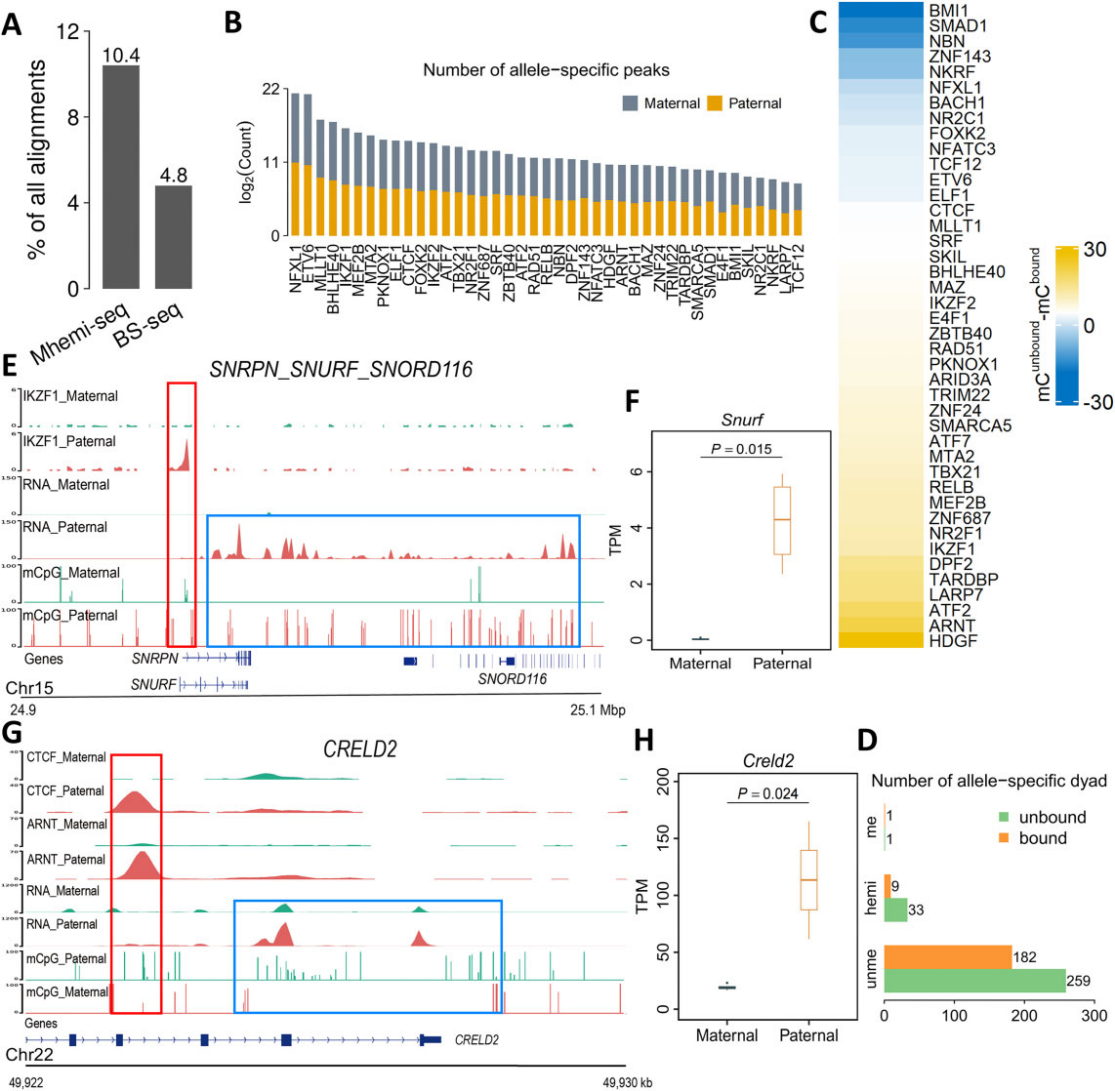
Mhemi-seq efficiently resolves allele-specific methylation and imprinted gene expression. **(A)** Comparison of SNPs captured by Mhemi-seq and BS-seq. **(B)** Number of maternal and paternal specific peaks of TFs in GM12878 cells. **(C)** Difference of methylation between unbound and bound allele of allele-specific peaks. **(D)** Mhemi-seq reveals the difference between unbound and bound allele of allele-specific peaks at CpG dyad level. **(E)** Enrichment of IKZF1 ChIP-seq, RNA-seq, and mCpG signals at SNRPN, SNURF, and SNORD116 genes. TF binding-associated and transcription-associated methylation are indicated by red and blue boxes, respectively. **(F**,**H)** Allele-specific expression of SNURF and CRELD2, respectively. The two-tailed *t*-test were performed on four RNA-seq datasets to determine whether the TPMs (transcript per million) at two alleles were significantly different. In addition, we applied Wilcoxon test (also known as Mann-Whitney test) to further validate the significance of the difference between TPMs on maternal and paternal alleles. **(G)** Enrichment of CTCF and ARNT ChIP-seq, RNA-seq and mCpG signals at CRELD2 gene.

In mammalian genome, the two alleles may exhibit allele-specific DNA methylation (ASM) patterns due to genomic imprinting or mutation, resulting in allele-specific gene expression (28,36,37). Previous studies have utilized BS-seq data to identify ASMs via single nucleotide polymorphisms (SNPs). However, BS-seq sacrifices a great portion of its capacity to identify SNPs due to C-to-T conversions introduced by bisulfite treatment (37–40). By contrast, Mhemi-seq avoids base conversion. Thus, we expected that Mhemi-seq can preserve more SNPs than BS-seq, allowing a more efficient study of ASM. To validate our assumption, we identified ASM using Mhemi-seq data in GM12878 cells whose parental genomes are sequenced and a comprehensive set of SNPs are well documented (41). Our data revealed that Mhemi-seq preserved >2-fold more SNP-containing fragments than BS-seq, and captured some ASM that BS-seq was unable to detect, indicating that Mhemi-seq may be a better tool for studying ASM than BS-seq (Supplementary Figure S3A and Figure 3A).

To further understand the role of ASM in transcriptional regulation, we investigated the correlation between ASM and allele-specific TF binding in GM12878 cells. We analyzed published ChIP-seq data of 59 TFs and found that many TFs had strong allele-specific binding, for example, NFXL1 has 3251 allele-specific ChIP-seq peaks (Figure 3B and C) (22). We then examined how ASM influences TF binding. Our data showed that the binding of most TFs was impaired by DNA methylation, and much less hemi-methylated CpG dyads were found in the allele-specific TF-bound regions than unbound regions (Figure 3D), indicating that hemi-methylation may play a similar role in inhibiting TF binding than full-methylation. By contrast, a few TFs, such as BMI1, preferred to bind the methylated alleles (Figure 3C). Allele-specific DNA methylation also instructs imprinted gene expression. BS-seq results showed that the promoters of imprinted genes normally have higher ASM levels (38,39,42). Here, using Mhemi-seq, we also identified ASM in imprinted genes. *SNRPN*, *SNURF* and *SNORD116* genes localize in an imprinted region and are paternally expressed (43). Our results showed that their paternal and maternal alleles were differentially methylated and bound by IKZF1, and only the paternal copy was expressed in GM12878 cells (Figure 3E, F and Supplementary Figure S3C). Besides imprinted genes, Mhemi-seq also identified previously unknown methylation-associated allele-specific gene expression (Figure 3G, H and Supplementary Figure S3B and S3C). For example, in GM12878 cells, a paternally expressed gene, *CRELD2*, exhibits paternal-specific low DNA methylation, CTCF and ARNT binding (Figure 3G and H). In summary, these results reveal that Mhemi-seq can efficiently preserve SNPs and identify ASM. Combining Mhemi-seq, ChIP-seq and RNA-seq data will help uncover the relationship between allele-specific DNA methylation, TF binding and gene expression.

### DNA hemi- and full-methylation exhibit similar degree of association with suppression of PRC1.1-associated genes

**Figure 4.**
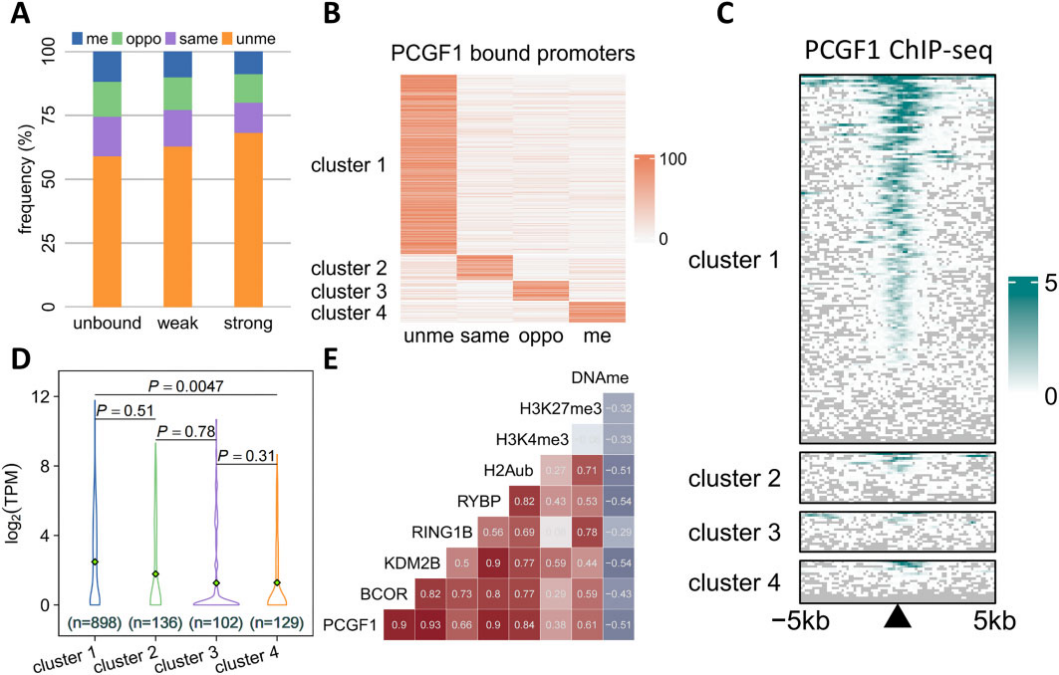
Both hemi- and full-methylation associate with PRC1.1-associated gene repression. **(A)** Frequency of four methylation statuses at PCGF1 unbound, weakly bound, and strongly bound promoters. me, oppo, same, and unme represent fully-methylated, opposite strand hemi-methylated, same strand hemi-methylated and unmethylated CpG dyads, respectively. **(B)** Promoters bound by PCGF1 could be divided into four types, according to their CpG dyad methylation status. **(C)** ChIP-seq signal of PCGF1 at four clusters of promoters bound by PCGF1. **(D)** Transcription levels at genes corresponding to the four clusters of promoters. Student's t test was used to calculate the significance *P*-value. **(E)** Pearson correlation of ChIP-seq and methylation signals over the promoters.

CpG hemi-methylation is very abundant in pluripotent cells (3), but whether hemi-methylation plays unique roles or just behaves like full-methylation is not clear. Polycomb repressive complex 1.1 (PRC1.1) is a key transcriptional regulator of ESCs, which associates with unmethylated CpG islands (CGIs) and actively transcribed genes (44–46). In addition, KDM2B, a subunit of PRC1.1, has been shown to inhibit hypermethylation in PRC1.1-bound regions (47). Therefore, we wonder whether DNA hemi-methylation can antagonize PRC1.1 to regulate transcription in ESCs. To address this question, we analyzed our Mhemi-seq data, and the ChIP-seq data of PCGF1 which is the core subunit of PRC1.1 (48), in hESC line H1. We found that PCGF1 prefers to bind promoter regions, and PCGF1-bound promoters have lower hemi- and full-methylation levels than unbound promoters (Figure 4A and Supplementary Figure S4A). To address how PCGF1 binding and DNA methylation influence gene expression, we grouped PCGF1-bound promoters into four clusters based on the methylation status of CpG in these promoters. Cluster 1, 2, 3 and 4 represent promoters in which most CpG were unmethylated, hemi-methylated at the sense or the antisense strand, and fully methylated, respectively (Figure 4B and Supplementary Figure S4B). Our data showed that PCGF1 binding and gene expression slightly anti-correlate with DNA methylation (Figure 4C and D). No significant difference was observed between gene expression at hemi-methylated and fully-methylated clusters (Figure 4D), suggesting that hemi-methylation may play a similar role of inhibiting gene expression as full-methylation.

To further understand how PRC1.1 influences the expression of cluster 1–4 genes, we analyzed the published ChIP-seq profiles of PCGF1, other PRC1.1 subunits, PRC-associated histone markers, and DNA methylation (Figure 4E and Supplementary Figure S4C). We found that the binding of PCGF1 was highly correlated with BCOR, KDM2B, and RYBP, while its correlation with RING1B, the E3 ligase that catalyzes H2A119ub, is lower (Figure 4E). As expected, we observed a moderately negative correlation between PRC1.1 binding and DNA methylation (Figure 4E). According to the correlation between PRC1.1 components and histone markers, PRC1.1 can be divided into two subtypes: (i) RING1B-dependent PRC1.1 that associates with the repressive marker H3K27me3 and (ii) RING1B-independent PRC1.1 that associates with the active marker H3K4me3. This indicates that PRC1.1 may have dual functions in H1 cells. In the presence of RING1B, PRC1.1 catalyzes H2AK119ub followed by H3K27me3 to repress gene expression, while in the absence of RING1B, KDM2B in PRC1.1 may keep genes active by inhibiting hypermethylation in promoters (49–51). Taken together, our work suggests that hemi-methylation is able to inhibit transcription of PCGF1-associated genes to the same extent as full-methylation. PRC1.1 can promote transcription via antagonizing with DNA methylation and repress genes in a RING1B-dependent manner. This dual function of PRC1.1 may play a pivotal role in gene regulation during embryonic development.

### Both hemi- and full-methylation on CTCF motifs inhibit CTCF binding

**Figure 5.**
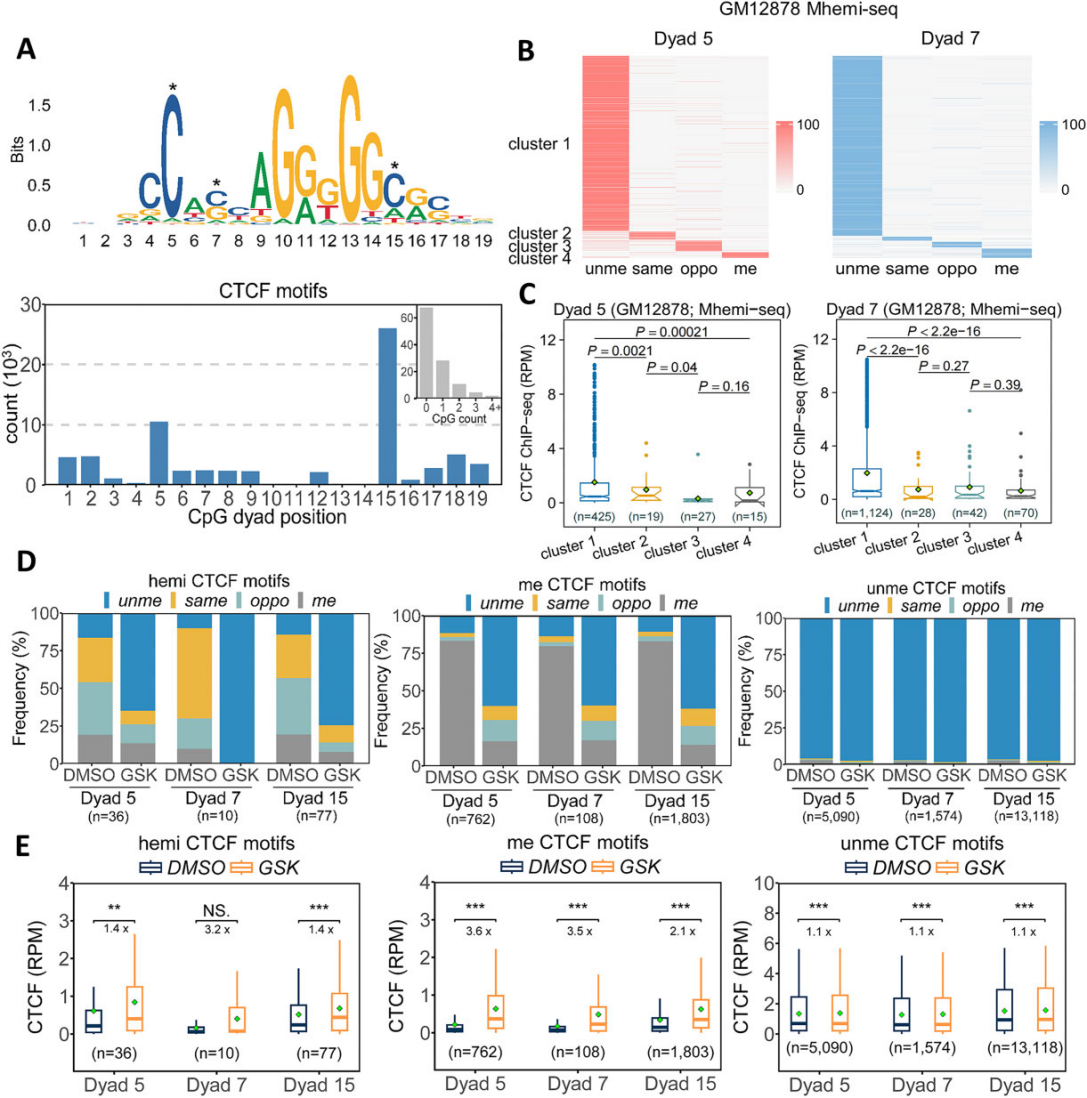
Both hemi- and full-methylation at CTCF motifs inhibit CTCF binding. **(A)** Top, the logo consensus motif. Three conservative cytosines (Dyad 5, 7, and 15) in CTCF motif are indicated by asterisks. Bottom, the number of motifs containing CpG at each position. **(B)** CTCF motifs could be divided into four types, according to the CpG dyad methylation status of cytosine in Dyad 5 or Dyad 7. Clusters 1–4 represent unmethylated, motif stand or opposite strand hemi-methylated, and fully-methylated CpGs, respectively. **(C)** CTCF ChIP-seq signal at motifs containing Dyad 5 or 7 in GM12878 cells. The circle on the box of boxplot denotes the mean value. **(D)** The frequency of four CpG methylation status at CTCF motifs containing hemi-methylated, fully methylated, or unmethylated Dyad 5, 7 or 15 in DMSO- or GSK-treated GM12878 cells. **(E)** CTCF ChIP-seq signal on motifs shown in panel (D) in DMSO- or GSK-treated GM12878 cells. Two-tailed Student's *t* test was used to calculate the significance *P*-value in this figure.

The conservative motif of CTCF harbors two frequent CpG dyads at position 5 and 15, and a few less frequent dyads at other positions (Figure 5A). An *in vitro* study showed that methylation at Dyad 5 in the CTCF motif significantly impaired CTCF binding, whereas the methylation at Dyad 15 did not (52). However, another *in vitro* study showed that hemi-methylation at both Dyad 5 and 15 on the motif strand significantly inhibited CTCF binding, while hemi-methylation on the opposite strand at Dyad 5 and 15 promoted CTCF binding (53). Conclusions from both studies were limited to single CTCF motifs, and thus, how cytosine methylation at different positions and strands influences CTCF binding is still not clear. To address this question, we analyzed the relationship between CTCF binding level and methylation status of dyads obtained from ChIP-seq and Mhemi-seq, respectively. Since the detection of methylation using Mhemi-seq relies on the YNCGNR sequence, here we focused on motifs with either Dyad 5 or 7 compatible with the YNCGNR sequence (Supplementary Figure S5A). To understand the role of different dyad methylation states, we grouped CTCF motifs into cluster 1, 2, 3 and 4 based on the methylation status of Dyad 5 or 7 (Figure 5B). We found that cluster 1 (unmethylated) was the dominant cluster, and the enrichment of CTCF at cluster 1 motifs were much higher than cluster 2 (motif strand hemi-methylated), 3 (opposite strand hemi-methylated), and 4 (fully methylated) motifs (Figure 5B, C, and Supplementary Figure S5B). This suggests that the binding of CTCF was significantly reduced by hemi- and full-methylation at both Dyad 5 and 7, which is consistent with the results using CpG dyads resolved by BS-seq and iSA (4) (Supplementary Figure S5C). In contrast to previous *in vitro* studies, our Mhemi-seq data showed that CTCF binding level is negatively correlated with Dyad 5 or 7 when fully methylated or hemi-methylated on either strand at either Dyad 5 or 7 in GM12878, H1, and H9 cells (Figure 5C and Supplementary Figure S5D), suggesting that hemi-methylation at Dyad 5 or 7 may block CTCF binding in a strand-independent manner in general.

To further understand how hemi-methylation regulates CTCF binding, we induced de-methylation in GM12878 cells using a newest-generation DNMT1 inhibitor, GSK-3484862 (referred to as GSK in this paper) (54,55). Since Mhemi-seq only captures hemi-methylation at CTCF motifs containing YNCGNR sequence, mostly at Dyad 7 positions (Supplementary Figure S5A), here we performed hpBS-seq to accommodate more CTCF motifs and more CpG dyad positions. In this analysis, we focused on methylation at Dyad 5, Dyad 7, and the most frequent dyad, Dyad 15 (Figure 5A). Upon GSK treatment, at a global level, 62.7% of fully methylated CpG dyads were converted to unmethylated dyads, while 72.0% of hemi-methylated CpG dyads were converted to unmethylated dyads (Supplementary Figure S5E). Consistent with a previous study, the global CTCF binding level was not significantly influenced by the reduction of DNA methylation level (Supplementary Figure S5F) (56). We next categorized CTCF motifs into three groups according to their predominant methylation status on Dyad 5, 7 or 15 (Supplementary Figure S5G), and investigated how methylation status changed for these dyads, and how CTCF binding level changed on the motifs harboring Dyad 5, 7 or 15, for each CTCF motif group. Upon GSK treatment, on Dyad 5, 7 or 15, both hemi- and full-methylation-predominant status transitioned to unmethylation-predominant status (Figure 5D). As expected, CTCF binding level on those ‘hemi-to-unme’ and ‘full-to-unme’ motifs were both significantly elevated (Figure 5E). Neither the methylation status nor the CTCF binding level changed on those unmethylation-predominant motifs (Figure 5D and E). The above results suggest that on some CTCF motifs, the hemi-methylation not only bookmarks the motifs, but also inhibits CTCF binding, and the inhibition can be relieved by inducing de-methylation.

### Mhemi-seq resolves CHG hemi-methylation on *Alu* elements

**Figure 6.**
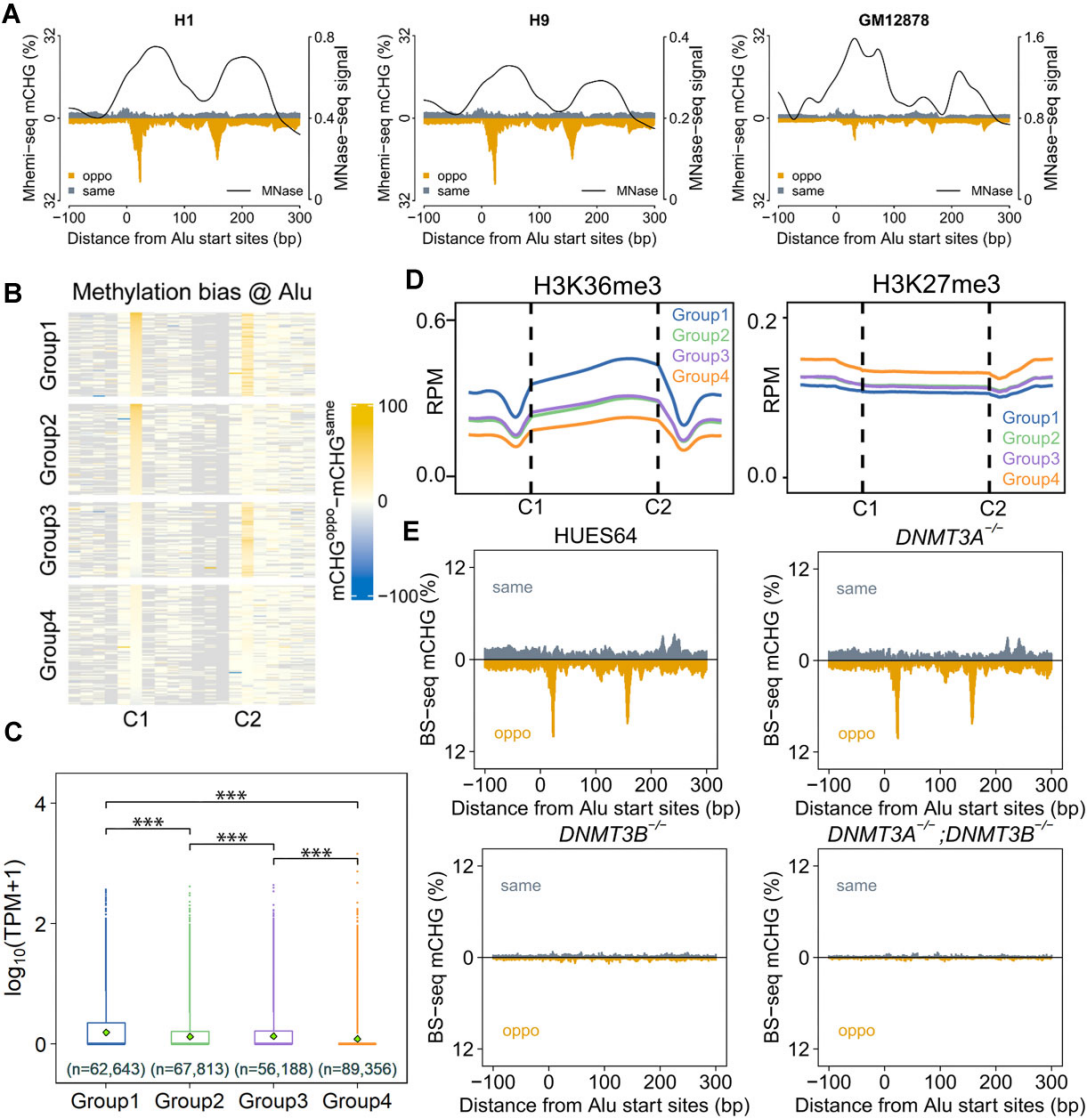
Mhemi-seq resolves CHG hemi-methylation on *Alu* elements. **(A)** Mhemi-seq resolves CHG methylation at the same (same, grey) and opposite strand (oppo, orange) of *Alu* elements in H1, H9 and GM12878 cells. MNase-seq signal is shown by the black curves. **(B)***Alu* elements are clustered into four groups based on the hemi-methylation at C1 and C2 in H1 cells. Group1 elements are hemi-methylated at both C1 and C2, while Group4 elements have no differential methylation at these two sites. **(C)** The hemi-methylation level at C1 and C2 correlates to *Alu* transcription. Student's t-test was used to calculate *P*-value. ***P< 0.001. **(D)** The enrichment of H3K36me3 and H3K27me3 on Group1-4 *Alu* elements. **(E)** CHG methylation on *Alu* elements in WT, DNMT3A knockout (DNMT3A-/-), DMMT3B knockout (DNMT3B-/-), and double knockout (DNMT3A-/-; DNMT3B-/-) HUES64 cell lines.

Besides CpG dyads, Mhemi-seq bears a similar principle to resolve methylation state of CpH (Figure 1A). In addition, similar to the YNCGNR motif, Mhemi-seq can retrieve the fragment length information to determine the methylation status of CHG dyads in the YCWGR (W = A/T) motif. To decipher the function of methylation at CHG sites, we first analyzed the methylation profiles at CHGR sites throughout the genome and found a special CHG methylation pattern in *Alu* retrotransposon elements. Due to its transposition activity, *Alu* elements are normally repressed in cells, but a subset of them can be re-activated in development and diseases (57–59). Previous work reveals that CpG methylation is critical for the repression of retrotransposons. However, the influence of CpH methylation on their repression is not clear. Our data reveals that *Alu* has a special methylation pattern in which the anti-sense strand of the *Alu* element is methylated at two CHG sites (C1 and C2), while the sense strand is poorly methylated in H1 and H9 cells (Figure 6A). Interestingly, we found that the methylation occurs close to the entry/exit site of the two nucleosomes on *Alu* elements (Figure 6A). By contrast, in GM12878 cells, this methylation pattern does not occur on *Alu* elements, and nucleosome phasing is fuzzy (Figure 6A). This suggests that the special methylation pattern correlates with nucleosome positioning on *Alu* elements. To understand why C1 and C2 are methylated, we analyzed the DNA sequence near these two CHG sites and discovered two similar consensus motifs with CTG at C1 and C2 (Supplementary Figure S6C). To confirm that these two sites were hemi-methylated, we analyzed the BS-seq results and found that both CTG dyads were indeed hemi-methylated at the CAG side (the reverse complement of CTG) (Supplementary Figure S6A and S6B). This suggests that the CHG hemi-methylation pattern may relate to the DNA context (60). To further understand the role of this hemi-methylation in *Alu* repression, we clustered *Alu* elements into four groups based on the differential methylation at C1 and C2 (Figure 6B). Our data reveals that the hemi-methylation at CAG positively correlated with the expression level of *Alu* (Figure 6B and C). Previous studies showed that cell type-specific *Alu* expression correlated with histone modifications on *Alu* elements (57,61). Here, to resolve the potential crosstalk between hemi-methylation and active histone markers, we analyzed the histone modification profiles of H1 cells. We found that the Group 1 *Alu* elements were enriched for an active transcription marker H3K36me3 (Figure 6D). Importantly, H3K36me3 is able to recruit the *de novo* methyltransferase DNMT3A/B to deposit DNA methylation, which may be the cause of hemi-methylation at CHG (62–64). To validate this hypothesis, we re-analyzed previously published BS-seq results of single and double knockout of DNMT3A and DNMT3B in HUES64 hESCs (65), and found that wildtype HUES64 exhibits the similar hemi-methylation pattern at *Alu* elements like H1 and H9. Interestingly, DNMT3B knockout abolished the strand-specific CHG methylation in *Alu*, while DNMT3A knockout did not (Figure 6E). This demonstrates that DNMT3B deposits hemi-methylation at CAG sites in *Alu* elements. Since DNMT3B can methylate CpG as well, we then checked the CpG methylation level at Groups 1–4 *Alu* elements. Similar to CAG methylation, the CpG methylation level on Group 4 Alu elements is lower than Groups 1–3 *Alu* (Supplementary Figure S6D). However, due to the high background CpG methylation, the change of CpG methylation is not as striking as CAG methylation. This suggests that DNA methylation may be context-dependent in regulating *Alu* repression. Compared to Group 1 *Alu* elements, Group 4 *Alu* elements were more enriched for H3K4me2/3 and H3K27me3, which are active and repressive markers, respectively (Figure 6D and Supplementary Figure S6E). These two markers normally co-exist on bivalent promoters, suggesting that the Group 4 *Alu* elements may be in a poised state (61). These findings are consistent with the gene expression profile, suggesting that the transcriptional activity of *Alu* associates with CAG hemi-methylation, nucleosome positioning, and histone modification. In summary, Mhemi-seq captures a special CHG hemi-methylation pattern in *Alu* elements, establishing a potential link between CHG hemi-methylation and transcription of *Alu* elements.

## Discussion

In this work, to uncover the function of hemi-methylation, we developed a restriction enzyme-based and bisulfite-free methylation mapping method, Mhemi-seq. In this method, methylated cytosines are detected by a methylation-dependent restriction enzyme, MspJI, which has been used in a few methylation mapping methods. However, the previous MspJI-based methods mainly focused on only capturing fragments with full-methylation or mapping strand-specific methylation in low resolution (12–14). Here, by retrieving fragments harboring only or both of full- and hemi-methylation, Mhemi-seq can efficiently resolve methylation status of single CpG and CWG dyads. Moreover, compared to other base-resolution methylation mapping methods, Mhemi-seq requires much less input DNA, costs less, and is easier to perform. In this study, using Mhemi-seq, we mapped the dyad methylomes of three cell lines, GM12878, H1, and H9, and investigated the roles of hemi-methylation in TF binding and gene expression.

How DNA methylation influences TF binding and downstream gene expression has been studied for decades. Past *in vitro* and *in vivo* studies have identified TFs that prefer to bind methylated or unmethylated motifs (28,66–69). However, how hemi-methylation, an abundant and unignorable component of DNA methylome, affects TF binding is not well understood. In this work, we discovered that hemi-methylation exhibits a broad spectrum of inhibitory effect on gene expression and TF-DNA binding. Some shows an inhibition in a less extent than full-methylation, whereas some displays a strong inhibition in the same degree as full-methylation. This may reflect different modes of how reader and effector proteins sense and translate DNA methylation marks. Some readers may work in an additive manner, translating hemi- or full-methylation into quantitively different downstream effects. On the other hand, some effector proteins may be completely blocked by hemi-methylation on either strand, resulting in indistinguishable inhibition caused by hemi- and full-methylation. Two recent reports discovered that some hemi-methylation can inhibit TF–DNA binding in a strand-specific manner, and achieved same degree of inhibition as full-methylation (53,70), suggesting that the binding of some TFs is only inhibited by methylation on a specific strand with respect to the motif, and is insensitive to the methylation state of the cytosine on the other strand. Interestingly, we discovered DNMT3B as the main methyltransferase responsible for depositing hemi-methylation on both CpG and CHG dyads in previous and present studies (3). DNMT3B is known to have a similar binding preference for unmethylated and hemi-methylated DNA (71). This renders two possible consequences for DNMT3B-catalyzed CpG: hemi-methylation or full-methylation, consistent with the similar degree of inhibition these two types of methylation exhibit on some CpG possibly through the strand-specific readers and effectors.

Besides the advantages described above, Mhemi-seq also has three limitations. Firstly, Mhemi-seq cannot map the whole methylome of a genome because MspJI recognizes a CNNR motif. For resolving CpG dyad methylation, the analysis of Mhemi-seq data is restricted to a YNCGNR motif, theoretically covering 25% of the CpG in the genome. This can be potentially circumvented in the future by the incorporation of other restriction enzymes from the MspJI family, many of which have a different recognition motif (11,14). Secondly, MspJI digestion at CGNA sites is not complete, resulting in a decrease of full methylation level. This deficiency does not influence the genome-wide study on methylation and hemi-methylation. However, it will impact the hemi-methylation level at single dyad resolution. To correct the bias caused by insufficient digestion at CGNA, we present a script which normalizes methylation level based on the cutting efficiency (see Materials and methods). Lastly, during library preparation, the 32 bp fragments harboring full-methylation and the long stretches of unmethylated fragments are hard to be captured, resulting in under-representation of extremely highly or poorly methylated regions in Mhemi-seq results. Thus, Mhemi-seq's advantages lie in its application in moderate to highly methylated genomes, especially when only a limited amount of starting materials are available, such as rare mammalian cell types or human biopsies for diagnosis, and when dyad resolution is preferred for DNA methylation analysis.

MspJI also recognizes a rare cytosine modification, 5hmC, which plays important role in gene regulation (14,72). Due to the very low abundance of this modification, the digestion at 5hmC has minor influence on the 5mC level in this work. Thus, we did not distinguish 5hmC from 5mC in our analysis. But by modifying the experimental procedures, we may be able to uncover 5hmC using Mhemi-seq. For example, the binding of MspJI to 5hmC can be blocked by T4 β-glucosyltransferase (T4 BGT) treatment that coverts 5hmC to 5ghmC, allowing the resolution of 5hmC from 5mC. This is a potential future direction of our work.

In summary, our data reveals that Mhemi-seq is a quantitative, sensitive, and cost-efficient method for DNA methylome analysis. By resolving methylation status of CpG and CHG dyads into unmethylation, strand-specific hemi-methylation, or full-methylation, Mhemi-seq presents a unique opportunity for dissecting the roles different states of DNA methylation play in chromatin activities. Due to its high sensitivity and amenability, we also look forward to the application of Mhemi-seq in disease diagnosis and high-throughput single-cell analysis of DNA methylomes in the near future.

## Supplementary Material

gkae023_Supplemental_Files

## Data Availability

All raw and processed data generated in this study can be obtained at Genome Sequence Archive (GSA) under accession number: HRA005249. Codes for Mhemi-seq data processing are available at https://github.com/xiongxionghhh/Mhemi-seq and https://doi.org/10.5281/zenodo.10453396.
