# Supplementary material for: Uncovering the roles of DNA hemi-methylation in transcriptional regulation using MspJI-assisted hemi-methylation sequencing
Source: Nucleic Acids Res. 2024 Jan 23;52(5):e24. doi: 10.1093/nar/gkae023 (PMC10954476; doi:10.1093/nar/gkae023)
Supplement: gkae023_Supplemental_Files [file gkae023_supplemental_files.zip › Mhemi_sup_figures_revised_chy_v4.pdf]

Supplementary Figure S1

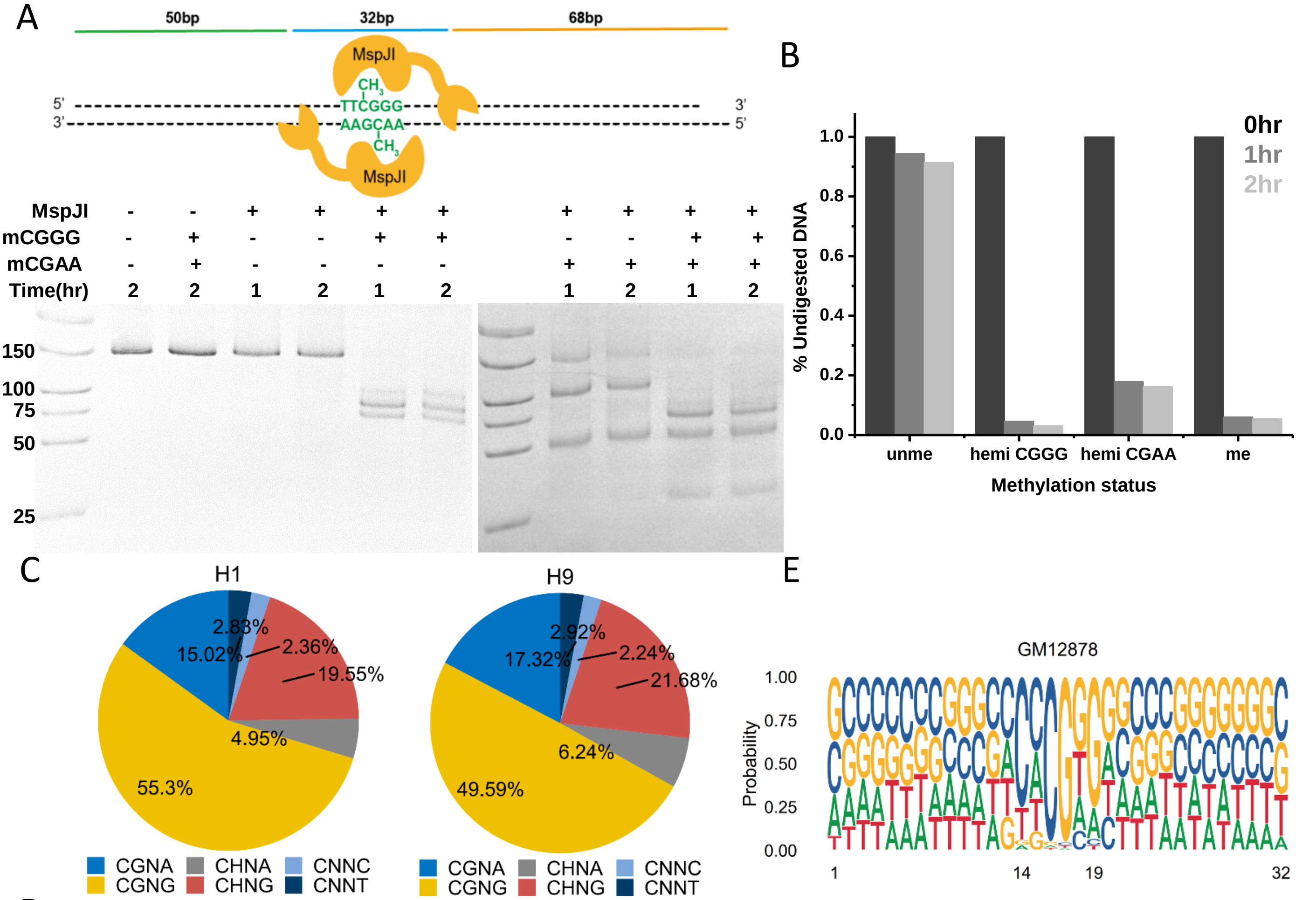

D

Cell line	Replicate	Raw reads (M)	Quality control (%)	Mapping (%)	MAPQ 10 (%)	Duplication (%)	Deduped alignment (M)	CGNR at both ends (%)	CpG (M)	CpG Dyad (M)	Efficiency dyad/alignment	Efficiency dyad/read
GM12878	1	61.5	97.4	94.7	92.8	11.0	46.9	66.1	129.3	16.4	0.35	0.27
	2	24.6	94.2	91.8	93.3	6.3	18.6	49.8	33.6	4.0	0.22	0.16
	3	19.4	94.6	93.3	93.1	5.7	15.0	53.5	30.5	3.7	0.25	0.19
H1	1	46.4	97.2	90.0	91.8	15.8	31.4	53.6	49.4	4.7	0.15	0.10
	2	30.8	93.5	91.9	92.2	16.7	20.3	54.0	34.1	3.8	0.19	0.12
	3	16.9	96.9	94.4	91.0	17.5	11.6	54.4	23.7	2.8	0.24	0.17
H9	1	48.8	98.2	90.6	91.8	12.8	34.8	51.1	58.1	6.0	0.17	0.12
	2	10.8	97.3	92.6	92.6	14.6	7.7	38.1	8.5	0.8	0.10	0.07
	3	59.7	97.2	94.4	92.3	22.2	39.3	46.9	56.1	5.5	0.14	0.09

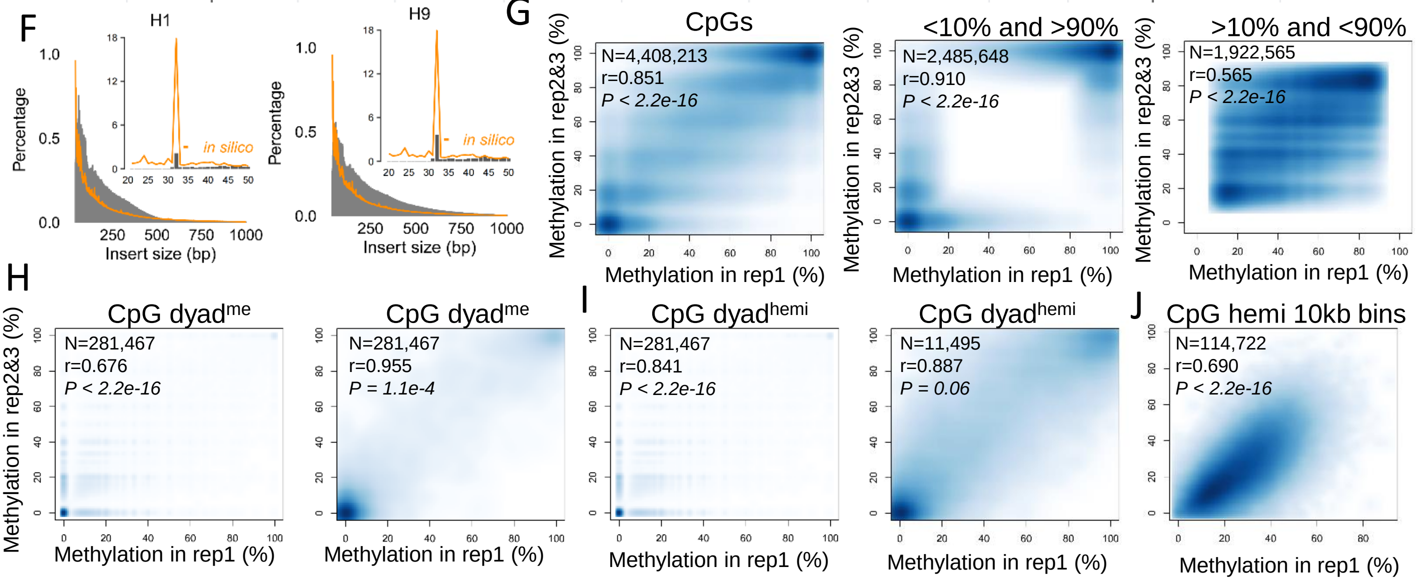

Supplementary Figure S1. Proof of principles and technical statistics of Mhemi-seq. **(A)** Assessment of MspJI digestion efficiency at mCGGG and mCGAA sites. DNA templates with an unmethylated, hemi-methylated, or fully methylated TTCGGG site were digested by MspJI for one or two hours. **(B)** Quantification of the panel S1B. The percentage of undigested DNA template was used to determine the cutting efficiency. **(C)** Motif analysis reveals the specificity of MspJI on CNNR sites in H1 and H9 cells. **(D)** Technical statistics of Mhemi-seq replicates. In this panel, “CGNR at both ends” is the indicator of MspJI cutting efficiency. An ideal probability of “CGNR at both ends” should be ~50%. **(E)** Logo of insert fragments in the length of 32 bp exhibits a conservative YNCGNR motif in position 14-19, which represents symmetrical YNmCGNR sites. **(F)** Length distribution of inserts in Mhemi-seq library (grey histogram) versus the length distribution simulated using BS-seq results (orange line). Experiments were performed with h1 and H9 cells. **(G)** Reproducibility of Mhemi-seq at CGNR sites. Methylation level of replicate 1 is plotted versus other replicates. Pearson’s correlation coefficient (r), *P*-value (*P*), and the number of CGNR sites (N) are shown on the plot. CGNRs with extreme (middle) and intermediate (right) methylation levels were plotted separately. **(H-I)** Reproducibility of Mhemi-seq at YNCGNR dyads. The proportion of methylated (me) CpG and hemi-methylation (hemi) at each YNCGNR dyad in replicate 1 are plotted versus their proportion in other replicates, respectively. YNCGNR sites covered by more than 5 (left) and 10 (right) reads are plotted. **(J)** The correlation of average hemi-methylation levels in 10kb bins.

Supplementary Figure S2

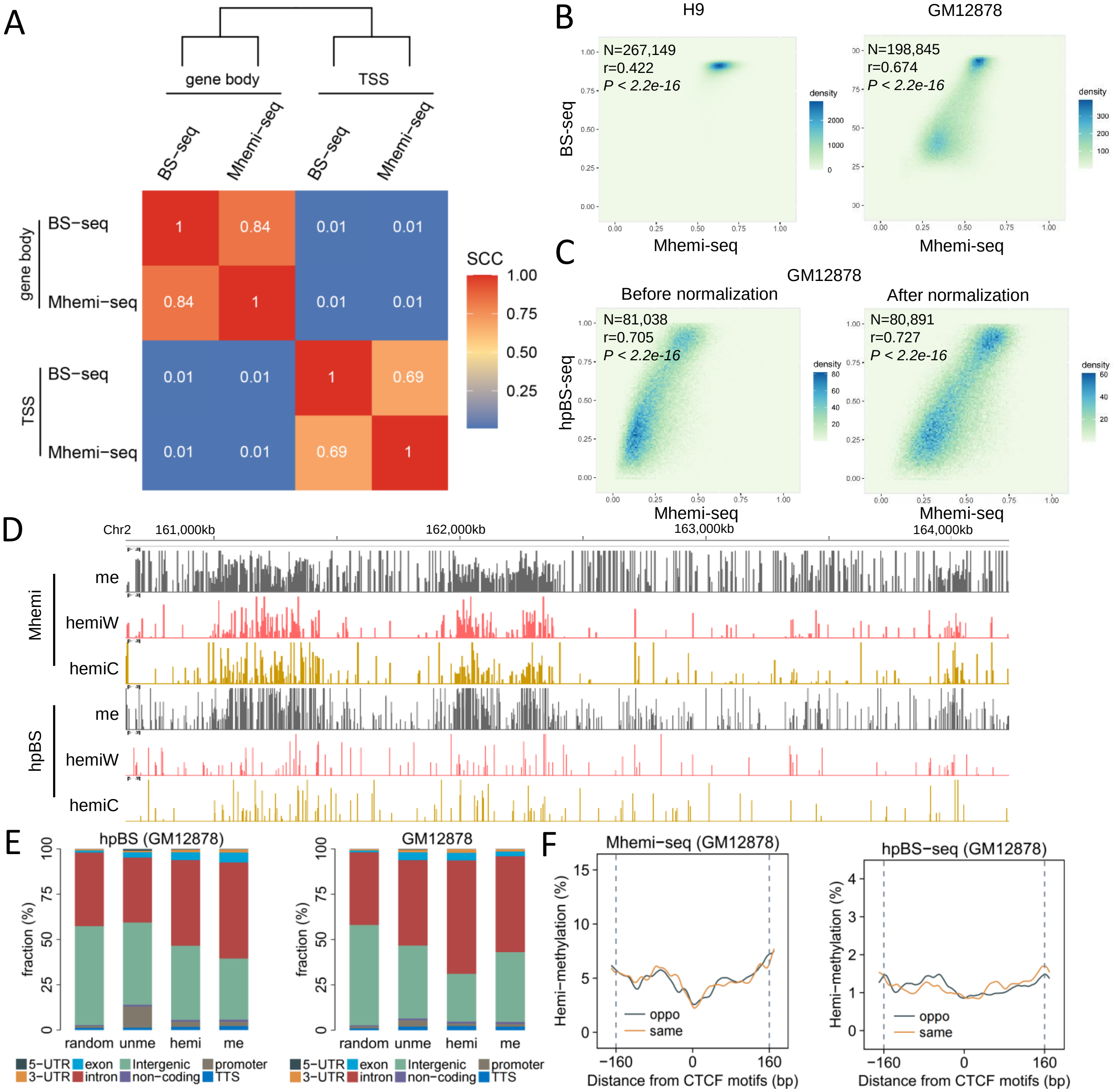

Supplementary Figure S2. Mhemi-seq reproduces cytosine methylome from BS-seq. **(A)** Spearman's correlation between Mhemi-seq and BS-seq at TSS and gene body in GM12878 cells. **(B)** CpG methylation levels in 10kb bins revealed by Mhemi-seq are plotted versus BS-seq results. Left, H9 cells. Right, GM12878 cells. N and r represent bin number and Pearson's correlation, respectively. Welch Two Sample t-test was used to calculate *P*-value. Bins containing less than 10 CpGs were removed from the plot. **(C)** Methylation levels at YNCGNR dyads revealed by Mhemi-seq are plotted versus hpBS-seq results. Left, raw Mhemi-seq data. Right, normalized Mhemi-seq data. **(D)** Genome browser tracks of methylation status at YNCGNR motifs captured by Mhemi-seq and hpBS-seq. me, full methylation; hemiW, hemi-methylation on Watson strand; hemiC, hemi-methylation on Crick strand. **(E)** The distribution of unmethylated, hemi-methylated, and fully-methylated CpGs in different genomic features. Left, hpBS-seq results. Right, Mhemi-seq results. **(F)** Strand-specific hemi-methylation profiles that Mhemi-seq and hpBS-seq mapped at CTCF motifs in GM12878 cells.

Supplementary Figure S3

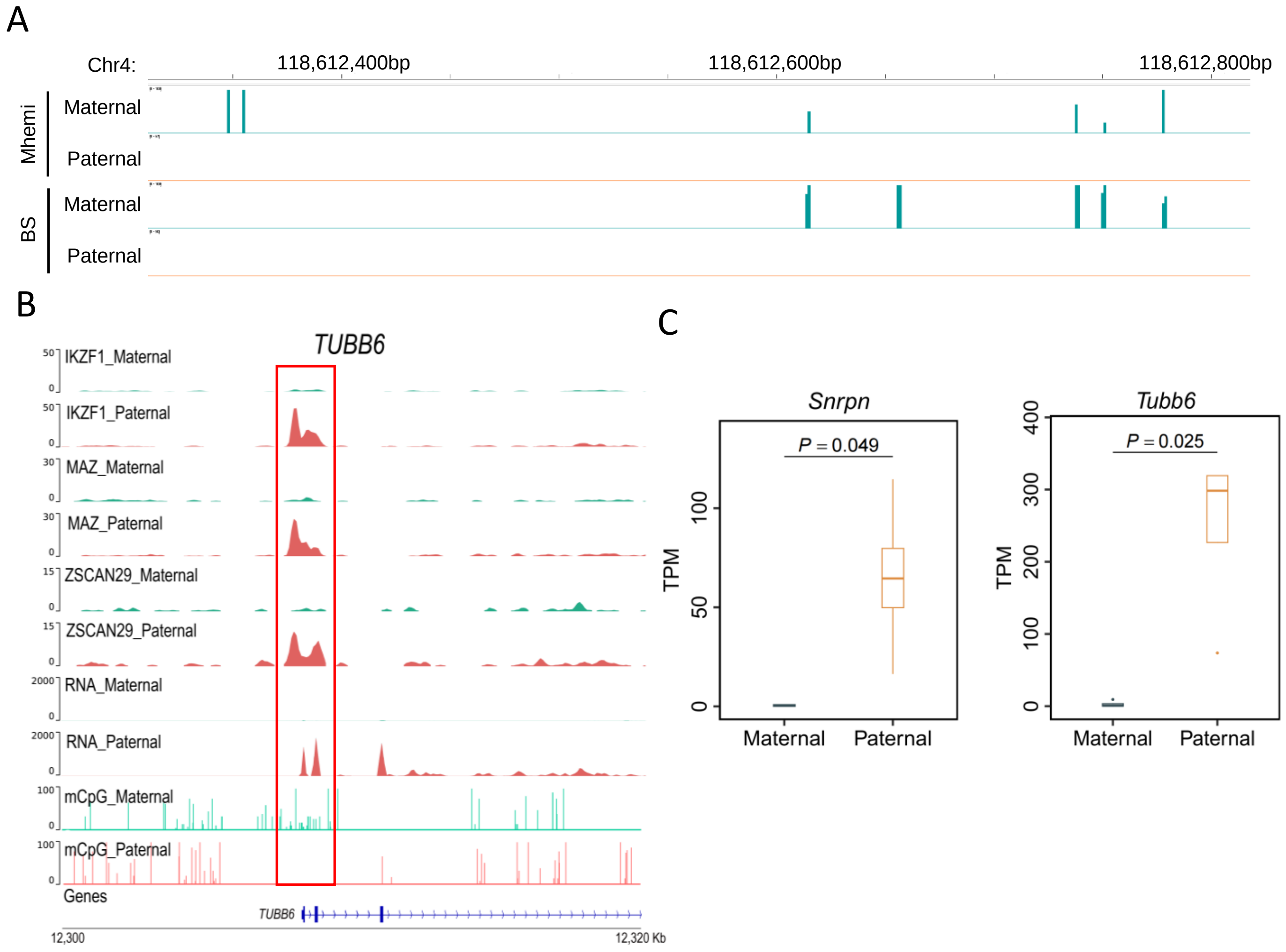

Supplementary Figure S3. Mhemi-seq efficiently resolves allele-specific methylation and imprinted gene expression. **(A)** Genome browser tracks of ASM captured by Mhemi-seq vesus BS-seq.**(B)** Enrichment of IKZF1, MAZ, ZSCAN29, RNA-seq, and mCpG signals at *TUBB6* gene. TF binding-associated and transcription-associated methylation is indicated by the red box. **(C)** Allele-specific expression of *SNRPN* and *TUBB6*, respectively. Student's t test was used to calculate the significance *P*-value.

Supplementary Figure S4

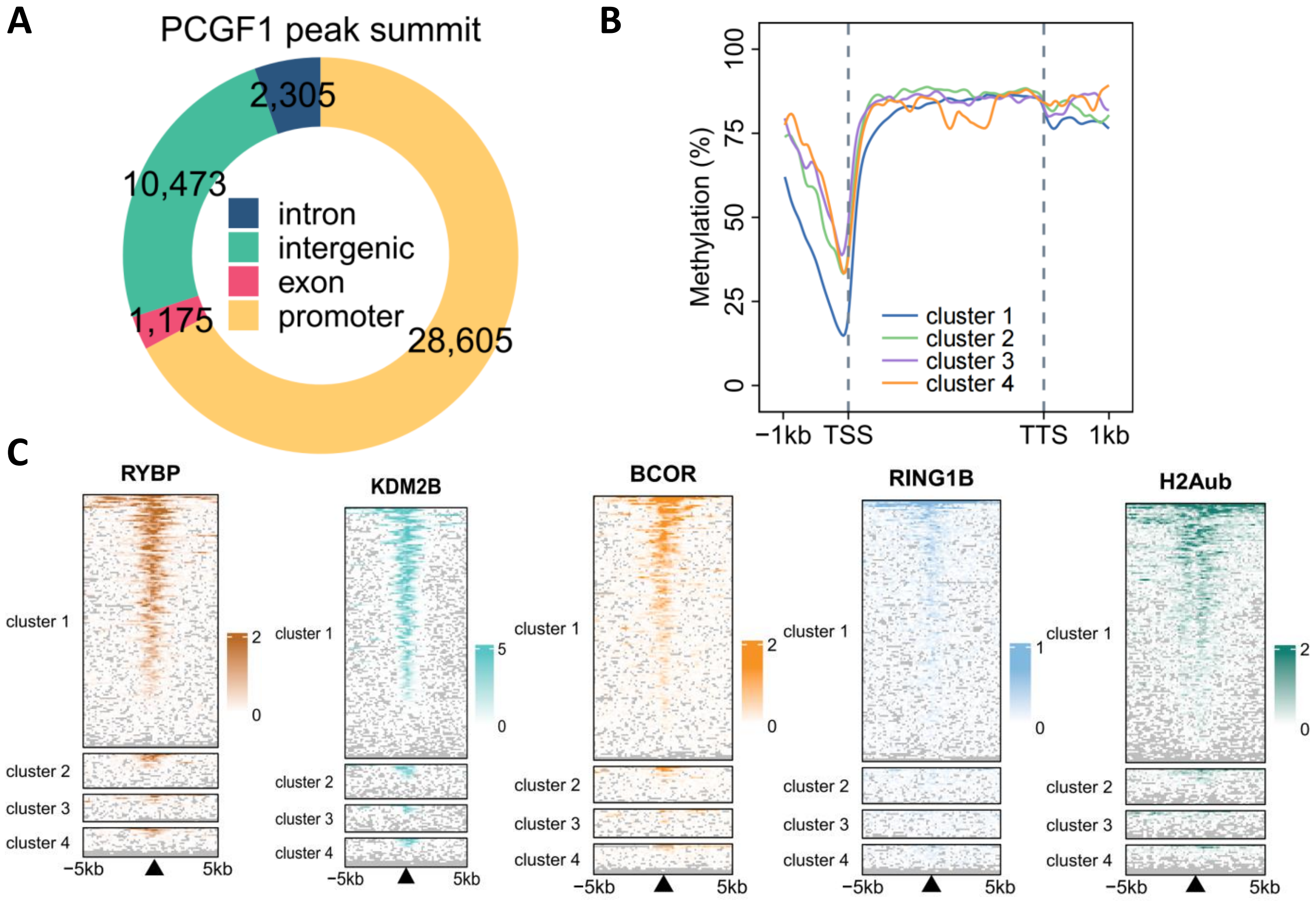

Supplementary Figure S4. Both hemi- and full-methylation associate with PRC1.1-associated gene repression. **(A)** PCGF1 peak summits are mainly located in promoters. **(B)** Average methylation levels at four clusters of PCGF1-bound promoters. **(C)** ChIP-seq signals of RYBP, KDM2B, BCOR, RING1B, and H2Aub at four clusters of PCGF1-bound promoters.

Supplementary Figure S5

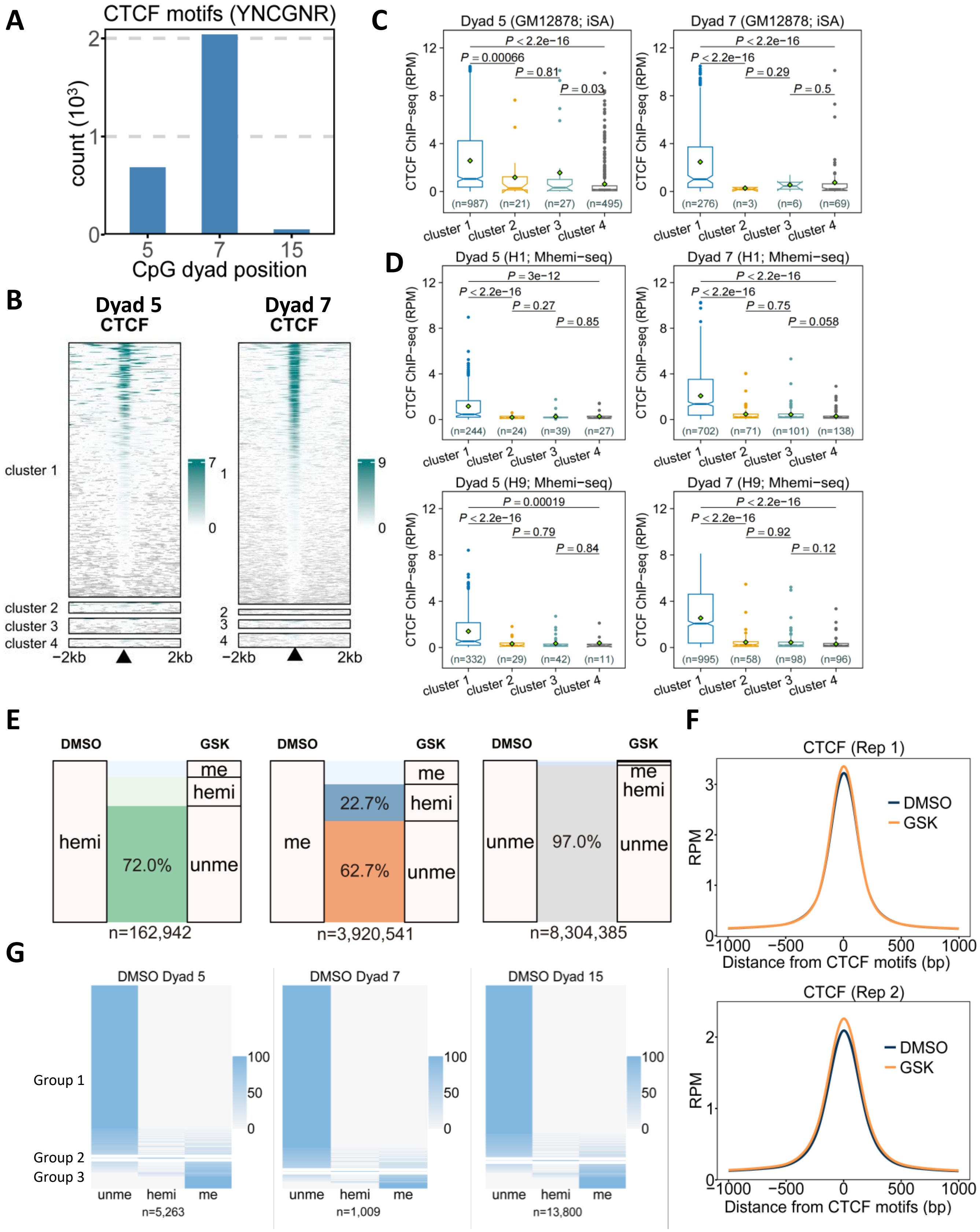

Supplementary Figure S5. Both hemi- and full-methylation at CTCF motifs inhibit CTCF binding. **(A)** Dyad 5 and Dyad 7 are in concordance with YNCGNR sequence. **(B)** CTCF ChIP-seq signal at CTCF motifs with different methylation status. Left, motifs with Dyad 5. Right, motifs with Dyad 7. **(C)** CTCF ChIP-seq signals of four clusters of motifs in GM12878 cells. Clusters are defined by BS-seq and iSA. **(D)** CTCF ChIP-seq signals of four clusters of motifs in H1 and H9 cells. Clusters are defined by Mhemi-seq results. **(E)** The change of three methylation statuses upon GSK treatment. Left, hemi-methylated CpGs. Middle, fully-methylated CpGs. Right, unmethylated CpGs. **(F)** CTCF ChIP-seq signals at CTCF motifs in DMSO (control, black) and GSK (orange) treated GM12878 cells. Top, experimental replicate 1. Bottom, experimental replicate 2. **(G)** CTCF motifs containing dyad 5, 7, or 15 are grouped into three clusters according to the methylation status at these three Dyads. Student's t test was used to calculate the significance *P*-value.

Supplementary Figure S6

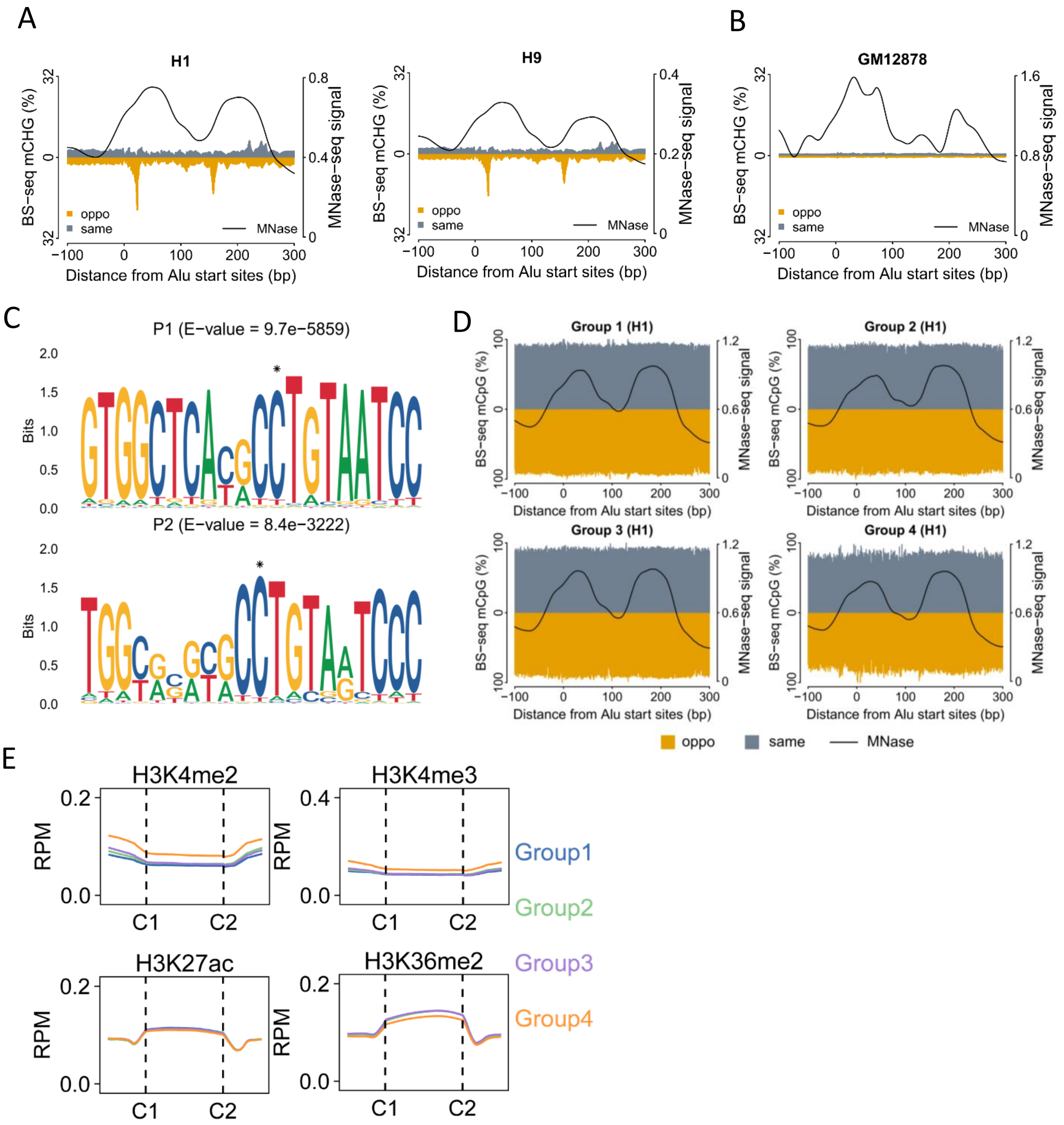

Supplementary Figure S6. Mhemi-seq resolves CHG hemi-methylation on *Alu* elements. **(A)** Mhemi-seq resolves methylation status at CHNR sites and CWG dyads. **(B)** CHG methylation at the same (same, grey) and opposite strand (oppo, orange) of *Alu* elements in H1, H9, and GM12878 cells. data collected by BS-seq. **(C)** Consensus sequences at hemi-methylated CHGs. **(D)** CpG methylation levels at group 1-4 *Alu* elements in H1 cells. **(E)** H3K4, H3K27, and H3K36 modifications at group 1-4 *Alu* elements.
