# Supplementary material for: Uncovering the roles of DNA hemi-methylation in transcriptional regulation using MspJI-assisted hemi-methylation sequencing
Source: Nucleic Acids Res. 2024 Jan 23;52(5):e24. doi: 10.1093/nar/gkae023 (PMC10954476; doi:10.1093/nar/gkae023)
Supplement: gkae023_Supplemental_Files [file gkae023_supplemental_files.zip › Supplementary File S1_protocol.docx]

**Mhemi-seq experiment**

1. Prepare a 20 μL reaction using 10-100 ng of genomic DNA, 10x NEB rCutSmart buffer, 30x MspJI activator, and MspJI (2.5 Unit per 100 ng of DNA.

**Note:** If you want to digest more DNA, scale-up the reaction instead of adding more DNA in the 20 μL reaction. See table below for recommended amount of enzyme. You may need to titrate the amount of enzyme to get the best digestion for your experiment.

**Recommended MspJI digestion conditions**

| DNA amount | MspJI | 45 μM adapter | PshAI digestion after PCR |
| --- | --- | --- | --- |
| 200 ng | 3-6 U | 1.5 | not needed |
| 100 ng | 2.5-6 U | 1.5 | not needed |
| 50 ng | 1.5-3 U | 1.0 | optional |
| 10 ng | 1-3 U | 0.5 | required |

**Note:** The activity of the enzyme may decrease during storage, so more enzyme may be needed.

1. Incubate the reaction at 37°C for one hour followed by the addition of 1 μL of 100 mM ATP (R0441, Thermo Fisher), 1.5 μL of 45 μM annealed Mhemi-seq adapter, and 400 Unit of NEB T4 Ligase. Incubate the reaction at 20-25°C for two hours.

**Note:** The amount of adapter needs to be adjusted according to the amount of genomic DNA as shown in table above.

**Critical:** **Do not increase incubation time to more than two hours!**

**Note:** The size of most digested fragments should be ~20-600 bp. 32 bp fragments will not be easily spotted on an agarose electrophoresis gel due to the low DNA amount.

1. Add 94 μL of AMPure beads (1:4) to the reaction, mix it by pipetting, and rotate it at 20-25°C for 10 min.

**Note:** The large volume of beads is used to capture the short target fragments (76 bp, 32 bp insert + two 22bp adapters).

1. Place tube on a magnetic rack and remove supernatant.
2. Wash beads twice with 80% ethanol.
3. Air-dry beads for 1-3 min to remove residual liquid, and then elute DNA from beads by 23.5 μL H_2_O.
4. Mix DNA sample with 25 μL of 2x KAPA HiFi HotStart ReadyMix (KK2602, Roche) and 1.5 μL of 10 μM sequencing primer mix.
5. Run this PCR reaction using the following cycling condition: 95°C 3 min for 1 cycle; 98°C 20 s, 65°C 10 s, and 72°C 5 s for 4-8 cycles; and 72°C 1 min for 1 cycle (Increase cycle number when using less input DNA).
6. Perform a test gel electrophoresis to check the amplified fragment size.

**Note:** A ~200-500 bp smear with a band at ~170 bp should be observed on an agarose gel as shown below. When input DNA amount is below 50 ng, you may see a strong adapter dimer band (dimer). In this scenario, PshAI digestion needs to be performed to remove excessive adapter dimers. No need to do PshAI digestion if the intensity of the dimer is weak.


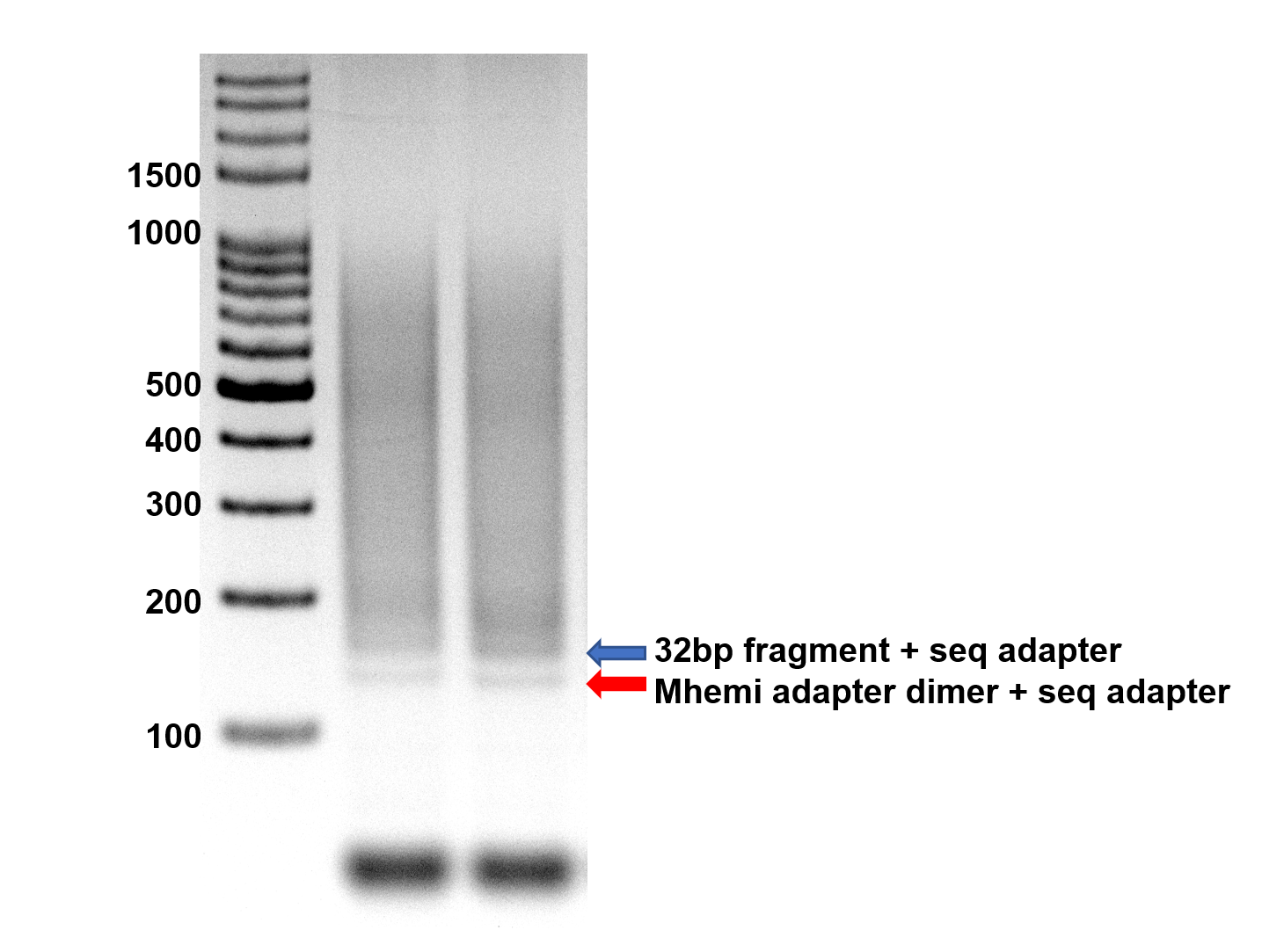


Fragment size of Mhemi-seq libraries

1. Clean-up PCR product using AMPure beads (1:1.4).
2. Sequence the libraries using Illumina NovaSeq 150 bp pair-end sequencing platform.

**Note:** The final amount of DNA should be ~20-50 ng. A small amount of adapter dimers in the final library is expected.

**Mhemi-seq data analysis**

1. Run pipeline_Mhemi.sh with raw paired-end sequencing reads in fastq format, and a reference genome sequence in fasta format.

**Note:** This script can be downloaded from <https://github.com/xiongxionghhh/Mhemi-seq/tree/main/Mhemi-seq/pipeline>.

**Critical:** This script requires the installation of bowtie2, fastp, trim_galore, samtools, and picard.

**Note:** If you used adapter containing PshAI cutting site, you need to run pipeline_Mhemi_PshAI.sh instead of pipeline_Mhemi.sh.

1. Read the files with names containing “_Report.txt”.

**Note:** Quality control information was saved in this file. The most important values in this file are “Motif (MspJI, 1bp wobble)” and “32bp”. Expected values are **~50%** and ~**5-6%**, respectively. These two parameters represent the amounts of fragments that contains CGNR at both ends and the fully methylated CpGs, respectively. They are affected by the MspJI digestion efficiency and the quality of library preparation.


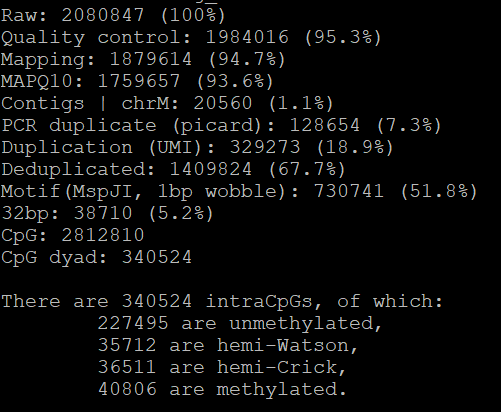


The screen shot of a Report.txt file.

1. Check the bed files in bed folder.

**Note:** The file contains “CpG_dyad.bed” in its name is the output for CpG dyad methylation. The seven columns in this file represents chromosome name, start position of CpG, end position of CpG, the count of unmethylated CpGs, the count of hemi-methylated CpGs at Watson stand, hemi-methylated CpGs at Crick strand, and fully-methylated CpGs.


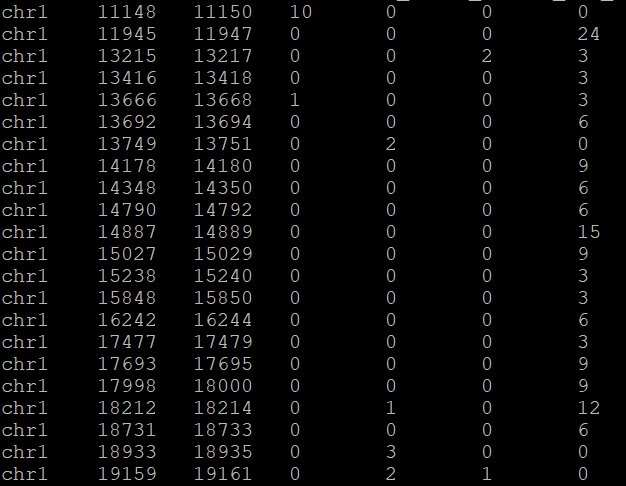


The screen shot of a CpG_dyad.bed file.

**Note:** The file contains “CpG.bed” in its name is the output for CGNR methylation. The six columns in this file represents chromosome name, start position of CpG, end position of CpG, the count of methylated CpGs, the count of all CpGs, and the strand of CGNR (+ represent Watson strand, while – represent Crick strand).


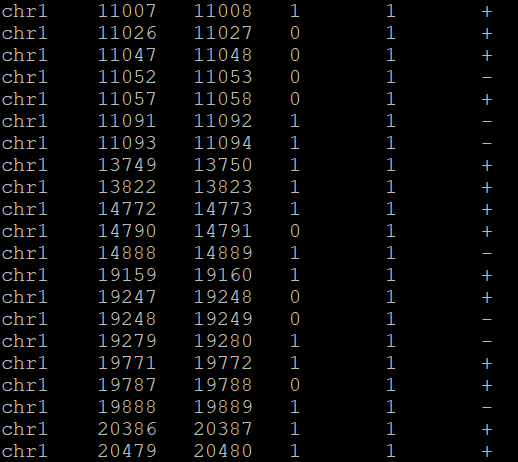


The screen shot of a CpG.bed file.

**Optional:** Normalize methylation information in CpG_dyad.bed using Mh_norm_v4.sh.

**Note:** Mh_norm_v4.sh can be downloaded from <https://github.com/HengyeChen/Mhemi_normalization>.

**Note:** This step is not necessary for genome-wide methylation analysis.

**Troubleshooting tips**

1. Insufficient digestion

If the genomic DNA is not digested enough, you can extend the digestion time to up to two hours or increase the amount of MspJI to 6 U.

1. Unable to capture 32 bp fragments in sequencing results.

Make sure you can see the band that represent the 32bp fragment on a gel or bioanalyzer after PCR. If you cannot see that band on the gel, you should try to increase the digestion time or ligation time.

1. Adapter contamination

Use Mhemi-seq adapter with PshAI cutting site and perform PshAI digestion after library preparation.

1. High or low “Motif (MspJI, 1bp wobble)” value

High “Motif (MspJI, 1bp wobble)” value indicates the genomic DNA is not digested enough, so more enzyme or longer digestion time is needed. In contrast, low value represents that DNA is under-digested, so you need to use less enzyme or reduce digestion time.
